# Comparison of the temperature- and pressure-dependent behavior of the crystal structure of CrAs

**DOI:** 10.1107/S2052520621005655

**Published:** 2021-07-23

**Authors:** Andreas Eich, Andrzej Grzechnik, Carsten Paulmann, Thomas Müller, Yixi Su, Thomas Wolf, Karen Friese

**Affiliations:** a Jülich Centre for Neutron Science-2 and Peter Grünberg Institute-4 (JCNS-2/PGI-4), Forschungszentrum Jülich GmbH, Jülich, 52425, Germany; b Institute of Crystallography, RWTH Aachen University, Jägerstraße 17-19, Aachen, 52066, Germany; c Mineralogisch-Petrographisches Institut, Universität Hamburg, Hamburg, 20146, Germany; d Jülich Centre for Neutron Science JCNS at Heinz Maier-Leibnitz Zentrum (MLZ), Forschungszentrum Jülich GmbH, Lichtenbergstraße 1, Garching, 85747, Germany; e Institute for Quantum Materials and Technologies, Karlsruhe Institute of Technology, Karlsruhe, 76021, Germany

**Keywords:** crystal structure, low temperature, high pressure, synchrotron diffraction

## Abstract

The crystal structure of CrAs was investigated using synchrotron X-ray single-crystal diffraction for separate dependences on temperature (30–400 K) and on pressure (0–9.46 GPa). The isosymmetrical magnetostructural phase transition at *T*_N_ = 267 K can induce a change in the microstructure by twinning due to a crossing of the orthohexagonal setting of the unit-cell parameter ratio *c*/*b*. Within the crystal structure, one particular Cr–Cr distance exhibits anomalous behavior in that it is nearly unaffected by temperature and pressure in the paramagnetic phase, which is stable above 267 K and at high pressures. The distinction of this shortest Cr–Cr distance might be of importance for the superconducting properties of CrAs.

## Abstract 2

The dependences of the crystal structure of CrAs on temperature (30–400 K) and on pressure (0–9.46 GPa) are reported. The investigation shows twinning accompanying the temperature-induced isosymmetrical phase transition and the existence of a distinct Cr–Cr distance within the structure showing anomalous behavior.

## 1. Introduction   

**Figure 1:**
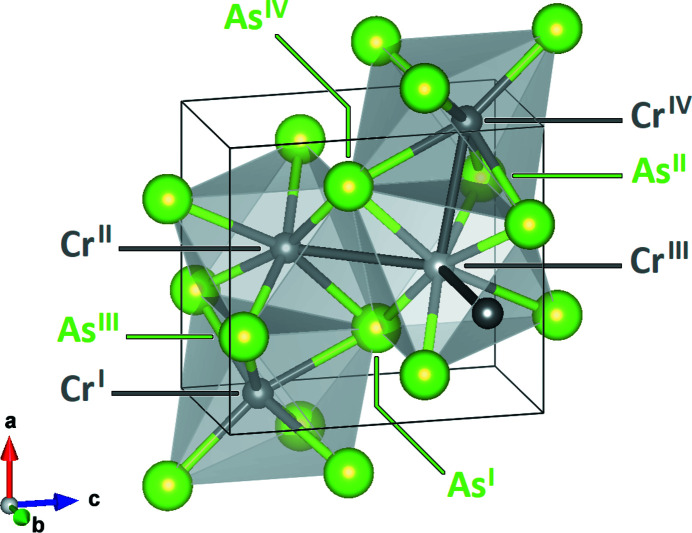
The crystal structure of CrAs. The Cr and As atoms are drawn in dark gray and green, respectively. The [CrAs_6_] octahedra are drawn in light gray.

Chromium arsenide (CrAs) crystallizes in the orthorhombic MnP-type structure (*Pnma*, *Z* = 4), commonly described as a distorted variant of the NiAs-type structure (*P*6_3_/*mmc*, *Z* = 2) (Fig. 1 ▸) (Rundqvist, 1962 ▸). The ideal NiAs-type structure can be understood as a hexagonal close-packed As sublattice interpenetrated by a primitive hexagonal Cr sublattice (Tremel *et al.*, 1986 ▸). The Cr atoms are sixfold coordinated by the As atoms in the shape of an octahedron [CrAs_6_]. The As atoms are sixfold coordinated by the Cr atoms in the shape of a trigonal prism [AsCr_6_]. In the MnP structure, both the [CrAs_6_] octahedra and the [AsCr_6_] prisms are distorted. The [CrAs_6_] octahedra form face-connected columns along the *a*-axis, which in turn are connected by shared edges. The [AsCr_6_] prisms are edge-connected forming a three-dimensional framework (Fig. 1 ▸).

At about 1170 K, a structural phase transition in CrAs from the ambient MnP to the parent NiAs structure is assumed based on the unit-cell parameter behavior (Selte & Kjekshus, 1973*a* ▸), but not confirmed by a full structure determination. The hexagonal NiAs-type structure can be described in an orthohexagonal setting in space group *Cmcm*, with *a*′ = *a*_hex_, *b′* = 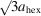 and *c*′ = *c*_hex_. A slight displacement of the atomic positions leads to the loss of the *C*-centering and the symmetry reduction to space group *Pmcn* following the *P*6_3_/*mmc*→*Cmcm*→*Pmcn* group–subgroup relation (Tremel *et al.*, 1986 ▸). Interchange of the axes leads to the standard setting *Pnma*. In this sequence of structural phase transformations, the threefold rotation axis is lost but indirectly preserved through the formation of three twin domains related by the threefold rotation axis along the *a*-axis of the lower symmetrical *Pnma* structure.

At *T*_N_ ≃ 267 K, CrAs undergoes a first-order magnetic phase transition from the room-temperature paramagnetic phase to a low-temperature helimagnetically ordered phase (Watanabe *et al.*, 1969 ▸), in which the Cr atoms carry a magnetic moment of ∼1.7 μ_B_ with all spins lying in the *ab* plane (Selte *et al.*, 1971 ▸). The magnetic moments propagate helically along the *c* axis with an incommensurate propagation vector **k** ≃ 0.354c* (Watanabe *et al.*, 1969 ▸). Due to the fact that the four helices can be separated into two in-phase pairs with a fixed angle between them, this structure is also described as double helical (Chen & Wang, 2019 ▸). The magnetic transition is accompanied by an abrupt change in the unit-cell parameters arguably without any symmetry change (Boller & Kallel, 1971 ▸). With Δ*a*/*a* ≃ −0.3%, Δ*b*/*b* ≃ +3.3% and Δ*c*/*c* ≃ −0.9% upon cooling from 268 K to 266 K, this leads to a large increase in unit-cell volume of Δ*V*/*V* ≃ +2.1% (Suzuki & Ido, 1993 ▸). Comparable changes in volume are also observed in other related compounds [*e.g.* Δ*V*/*V* ≃ +2.1% in MnAs (Suzuki & Ido, 1982 ▸; Motizuki *et al.*, 2009 ▸)].

Phase transitions in general are a common feature of transition-metal pnictides. The majority of these compounds crystallize in either the MnP or the NiAs structure type (Motizuki *et al.*, 2009 ▸; Boller & Nowotny, 1965 ▸). Table 1 ▸ gives an overview of the compounds that exhibit the MnP-type structure (Kazama & Watanabe, 1971 ▸) including the observed phase transitions and the available structural data.

Compounds CrAs, CoAs (Heyding & Calvet, 1957 ▸) and MnAs (Wilson & Kasper, 1964 ▸) exhibit a transition between the two structure types, though a theoretical NiAs→MnP transition at high temperatures as proposed for CrP (Selte *et al.*, 1975 ▸) might be a more general phenomenon in these compounds. Isosymmetrical phase transitions^1^ are reported both between two NiAs-type structures [CrSb (Suzuki & Ido, 1986 ▸), MnSb (Willis & Rooksby, 1954 ▸) and MnBi (Willis & Rooksby, 1954 ▸)] and between two MnP-type structures [MnP (Fjellvåg & Kjekshus, 1984 ▸; Zhigadlo *et al.*, 2017 ▸), FeP (Westerstrandh *et al.*, 1977 ▸; Chernyavskii *et al.*, 2020 ▸) and FeAs (Selte *et al.*, 1972*a* ▸; Selte & Kjekshus, 1973*b* ▸)]. In the latter compounds, including CrAs, the isosymmetrical MnP→MnP transition is coupled to the onset of magnetic order. The magnetic structure at low temperatures is consistently a helimagnetically ordered phase with the helix propagating along the *c* axis, with only MnP showing in addition an intermediate ferromagnetic phase. While the MnP→MnP transitions in FeP and FeAs and the high-temperature one in MnP (from a para- to the ferromagnetic state) are of second order, the low-temperature transitions in MnP (from the ferro- to the helimagnetic state) and in CrAs are of first order and accompanied by abrupt changes in the unit-cell parameters, though the precise changes in MnP are controversial (Shapira *et al.*, 1981 ▸; Okamoto *et al.*, 1968 ▸). MnP and CrAs show a further similarity as, for these two compounds, pressure-induced superconductivity has been confirmed (Chen & Wang, 2019 ▸). Although the current lack of systematic structural data for MnP prohibits a conclusive analysis, the observed similarities might indicate a more general connection between the structural and the superconducting properties of the transition-metal pnictides.

In CrAs, the changes in both the magnetic properties and the crystal structure are related to the underlying electronic structure. Zavadskii & Sibarova (1980 ▸) proposed an electronic model for CrAs, explaining the coupled magnetic and structural behavior. In this model, covalent Cr—As bonds and strong Cr–Cr interactions are considered. The coupling of the spins on the Cr atoms is antiferromagnetic and the incommensurate and non-collinear magnetic structure is assumed to be a result of complex interactions between different Cr atoms (Selte *et al.*, 1971 ▸). Above *T*_N_, CrAs exhibits Pauli paramagnetism (Selte *et al.*, 1971 ▸; Zavadskii & Sibarova, 1980 ▸), where only the unpaired valence electrons near the Fermi level contribute to the magnetic moment. The strong correlation of electronic, magnetic and structural properties makes CrAs highly sensitive to hydro­static compression. According to *ab initio* calculations, the *Pnma* phase of CrAs undergoes a pressure-induced phase transition to a cubic phase (*P*2_1_3) at about 33 GPa at very low temperatures (Wang *et al.*, 2017 ▸), though this transition has not been observed experimentally so far. The same transition *Pnma*→*P*2_1_3 has been predicted for FeP at 88 GPa (Yan, 2015 ▸).

On increasing pressure, the transition temperature *T*_N_ decreases with an initial gradient d*T*_N_/d*p* of about −18 K kbar^−1^ (−180 K GPa^−1^) (Zavadskii & Sibarova, 1976 ▸), with a steepening decrease at higher pressures and an eventual complete suppression of the magnetic transition above a critical pressure *p*_c_ ≃ 0.7 GPa (Kotegawa *et al.*, 2014 ▸; Matsuda *et al.*, 2018 ▸). This can be understood as an increasing stabilization of the low-volume phase with pressure.

The second effect of hydro­static pressure on CrAs is the induction of superconductivity. Chromium arsenide is the first Cr-based compound in which superconductivity was discovered (Kotegawa *et al.*, 2014 ▸). Above ∼0.3 GPa (Wu *et al.*, 2014 ▸; Shen *et al.*, 2016 ▸), CrAs exhibits a dome-like-shaped superconducting phase region with a maximum *T*_c_ ≃ 2.2 K at about 1.0 GPa (Kotegawa *et al.*, 2014 ▸). Between the onset of superconductivity at 0.3 GPa and the suppression of the magnetic phase at 0.7 GPa, a two-phase region with competing magnetic and superconducting properties is observed (Keller *et al.*, 2015 ▸; Khasanov *et al.*, 2015 ▸). The nature of the superconductivity is not yet understood (Cheng & Luo, 2017 ▸), with different results supporting both conventional (Khasanov *et al.*, 2015 ▸) and unconventional (Wu *et al.*, 2014 ▸; Kotegawa *et al.*, 2015 ▸; Shen *et al.*, 2016 ▸; Nigro *et al.*, 2019 ▸) pairing mechanisms. In the case of the unconventional superconductivity, the pairing is assumed to be mediated by antiferromagnetic fluctuations between nearest-neighbor Cr atoms (Chen & Wang, 2019 ▸; Shen *et al.*, 2016 ▸). Due to this, CrAs is considered to be a model system to study the interplay of unconventional superconductivity and helimagnetic order (Cheng & Luo, 2017 ▸).

Up to now, most studies on CrAs in and near the superconducting phase region have been focused on its magnetic (Wu *et al.*, 2014 ▸; Khasanov *et al.*, 2015 ▸; Kotegawa *et al.*, 2015 ▸; Matsuda *et al.*, 2018 ▸) and transport (Kotegawa *et al.*, 2014 ▸; Wu *et al.*, 2014 ▸; Kim *et al.*, 2017 ▸) properties. The crystal structure of CrAs, however, has not been thoroughly investigated as a function of temperature and little is known about the exact evolution of structural parameters above and below *T*_N_. The temperature-dependent measurements reported in the literature mostly concern the unit-cell parameters, and only a few isolated data points exist for the full characterization of the crystal structure (Nowotny & Årstad, 1938 ▸; Selte *et al.*, 1971 ▸; Kazama & Watanabe, 1971 ▸; Boller & Kallel, 1971 ▸; Keller *et al.*, 2015 ▸; Shen *et al.*, 2016 ▸; Sen *et al.*, 2019 ▸). A powder diffraction study of the high-pressure behavior of the crystal structure at room temperature (Yu *et al.*, 2015 ▸) shows anomalies in the unit-cell parameters at about 0.3 GPa, indicating an isosymmetrical phase transition. This transition is proposed to result from a change in the electronic structure. It is noted that it coincides with the critical pressure corresponding to the onset of superconductivity at low temperature. The aim of the present work is to present a detailed characterization of the crystal structural behavior of CrAs at different temperatures (35–400 K) and on compression to 9.5 GPa to elucidate the role of the strong Cr–Cr interactions. These investigations will also serve as reference for future combined pressure–temperature studies in and near the superconducting phase region where the coupling of structure and superconductivity can be studied directly. Furthermore, this study of CrAs is a first step to instigate a more systematic investigation of the role of the crystal structure in the various properties of the transition-metal pnictides, *e.g.* the rather similar MnP.

## 2. Experimental   

### 2.1. Synthesis   

Single crystals of CrAs were grown using the Sn-flux method described by Wu *et al.* (2010 ▸). The crystals are needle-shaped with sizes approximately 7 mm × 1.5 mm × 1.5 mm, grown along the *a* axis. The crystal habit is similar to a hexagonal system, which can be explained as a result of the previously mentioned transition from the hexagonal to the orthorhombic structure (Sen *et al.*, 2019 ▸).

### 2.2. Laboratory X-ray diffraction   

Temperature-dependent X-ray diffraction measurements in the range 240–400 K were performed on a Rigaku Oxford Diffraction SuperNova single-crystal diffractometer with a Mo *K*α X-ray source. The temperatures were set using an Oxford Instruments nitro­gen flow gas jet cryostat/heater. Diffraction data were integrated with the *CrysAlisPRO* (v171.40.53; Rigaku Oxford Diffraction, 2019 ▸) program and the structure refinements were carried out with *Jana2006* (Petříček *et al.*, 2014 ▸).

Exploratory high-pressure X-ray diffraction measurements to 7.3 GPa at room temperature were performed on a Stoe IPDS-II single-crystal diffractometer with a Mo *K*α X-ray source using a Boehler Almax diamond anvil cell loaded with a CrAs crystal, a ruby chip and a 4:1 methanol–ethanol mixture as a pressure-transmitting medium. The diffraction data were integrated with the *X-Area* (v1.62; Stoe & Cie, 2011 ▸) software.

### 2.3. Synchrotron X-ray diffraction   

The synchrotron diffraction experiments were performed on beamline P24 at PETRA III (DESY, Hamburg, Germany) on the kappa diffractometer (EH1, λ = 0.45085 Å) equipped with a Pilatus CdTe 1M area detector.

For the measurements in the range 35–275 K at ambient pressure, the temperatures were set using a Cryocool G2b-LT helium gas jet cryostat.

The high-pressure measurements to 9.5 GPa at room temperature were carried out using several diamond anvil cells, each pre-loaded with a CrAs single crystal, a ruby chip, and a 4:1 methanol–ethanol mixture as pressure-transmitting medium. The pressure during all measurements in diamond anvil cells was determined using the ruby luminescence method (Mao *et al.*, 1986 ▸).

All synchrotron diffraction data were integrated with the *CrysAlisPRO* (v171.40.53; Rigaku Oxford Diffraction, 2019 ▸) software and subsequently refined with the *Jana2006* (Petříček *et al.*, 2014 ▸) program. The equation of state was determined using *EosFit7* software (Angel *et al.*, 2014 ▸; Gonzalez-Platas *et al.*, 2016 ▸).

Detailed information on the laboratory and synchrotron measurements and on the refinements are given in Tables S1–S10.

## 3. Results   

### 3.1. Symmetry considerations   

For all measurements (synchrotron and laboratory), the observed diffraction patterns exhibit extinction rules that are in accordance with space groups *Pnma* and *Pn*2_1_ *a*. Although the intensity statistics hint towards the acentric *Pn*2_1_ *a* structure, refinements in both space groups (including an inversion twin model in space group *Pn*2_1_ *a*) showed no significant difference in the overall quality of the fit. However, refinement of the parameters *y*_Cr_ and *y*_As_, which are fixed to ¼ in *Pnma* but free in *Pn*2_1_ *a*, show that within the errors they do not deviate from the centrosymmetric value ¼ in the whole temperature range. This observation is similar to the findings of Selte & Kjekshus (1973*b* ▸) regarding the crystal structure of the isostructural compound FeAs. Based on these observations, the space group *Pnma* was assumed to be the correct choice for CrAs at all conditions.

### 3.2. Unit-cell parameters as function of temperature   

**Figure 2:**
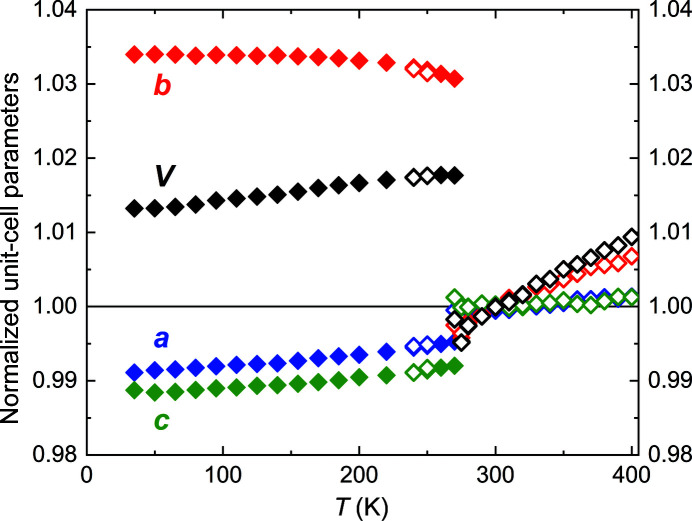
Unit-cell parameters and unit-cell volume normalized to their respective values at room temperature as a function of temperature. Data from synchrotron and laboratory X-ray measurements are indicated by filled and open symbols, respectively.

The dependence of the unit-cell parameters on temperature – normalized to the value at room temperature – is shown in Fig. 2 ▸. Above the phase transition, the observed behavior of the unit-cell parameters agrees quite well with the high-temperature data measured by Selte & Kjekshus (1973*a* ▸), and shows in particular the same tendencies. At *T*_N_, the structural phase transition is clearly visible from the large abrupt changes of all unit-cell parameters. The relative changes on cooling Δ*a*/*a* ≃ −0.40%, Δ*b*/*b* ≃ +3.51%, Δ*c*/*c* ≃ −0.83% and Δ*V*/*V* ≃ +2.25% confirm the previous observations from the literature. Below the transition temperature, the unit-cell parameters change smoothly; *a* and *c* decrease in a comparable way, *b* first increases and eventually stays nearly constant below about 200 K, where due to the lack of further change in *b*, the volume *V* is dominated by the behavior of *a* and *c* (Fig. 2 ▸).

**Figure 3:**
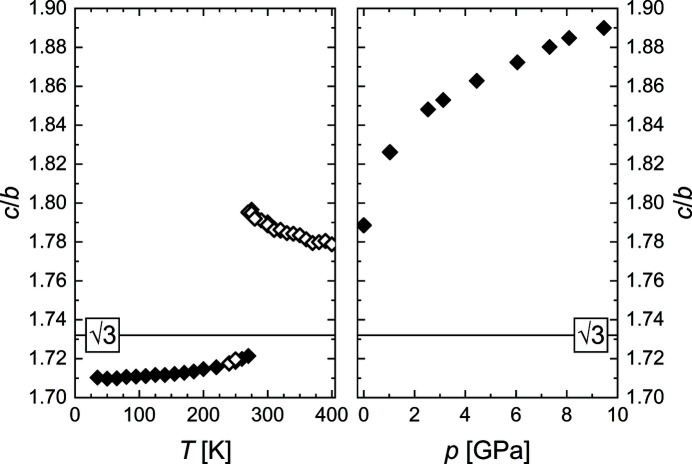
The *c*/*b* ratio as a function of temperature (left) and pressure (right). Data from synchrotron and laboratory X-ray measurements are indicated by filled and open symbols, respectively.

These drastically different behaviors of *b* and *c* result in an important change in the *c*/*b* ratio, shown in Fig. 3  ▸(left). This *c*/*b* ratio of the orthorhombic setting is related to the *a*_hex_/*b*_hex_ ratio of the hexagonal parent structure, and in the ideal orthohexagonal setting it is *c*/*b* = 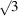 . Undergoing the phase transition, CrAs abruptly changes from *c*/*b* > 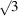 to *c*/*b* < 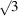 . While this change in the ratio has been noted before for CrAs (Selte *et al.*, 1971 ▸) and the ratio was used in a general sense to distinguish two subclasses of the MnP-type structure (Pfisterer & Schubert, 1950 ▸), no further significance was attributed to it. However, we believe that the fact that the *c*/*b* ratio crosses the ideal value of 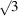 is crucial for a change in the twin domain structure observed during the transition, which will be discussed in detail below.

### 3.3. Unit-cell parameters as function of pressure   

**Figure 4:**
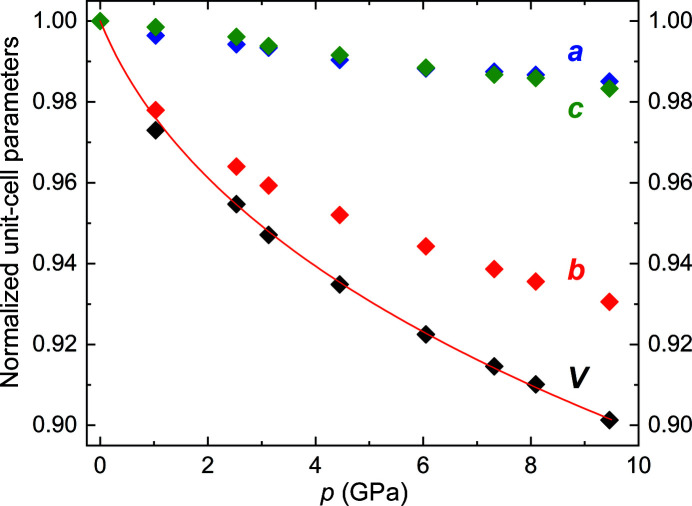
Unit-cell parameters and unit-cell volume normalized to their respective values at ambient pressure as a function of pressure. The line represents the fit of the third-order Birch–Murnaghan equation of state to the data.

In contrast to the behavior at low temperatures, the application of pressure does not lead to the first-order phase transition like the one at *T*_N_ (Fig. 4 ▸). This is easily understandable, as the low-temperature phase transition is coupled to an increase in volume and thus cannot be induced by increasing pressure. All unit-cell parameters decrease with pressure, with the *b* axis exhibiting a significantly higher compressibility than the *a* and *c* axes, which show approximately the same compressibility. The *c*/*b* ratio increases with pressure (Fig. 3 ▸, right), moving away from the ideal value of 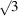 corresponding to the orthohexagonal setting. The same pressure-dependent behavior is observed in other transition-metal pnictides with the MnP-type structure, *e.g.* FeAs (Jeffries *et al.*, 2011 ▸), MnP (Han *et al.*, 2018 ▸) and FeP (Gu *et al.*, 2011 ▸).

**Figure 5:**
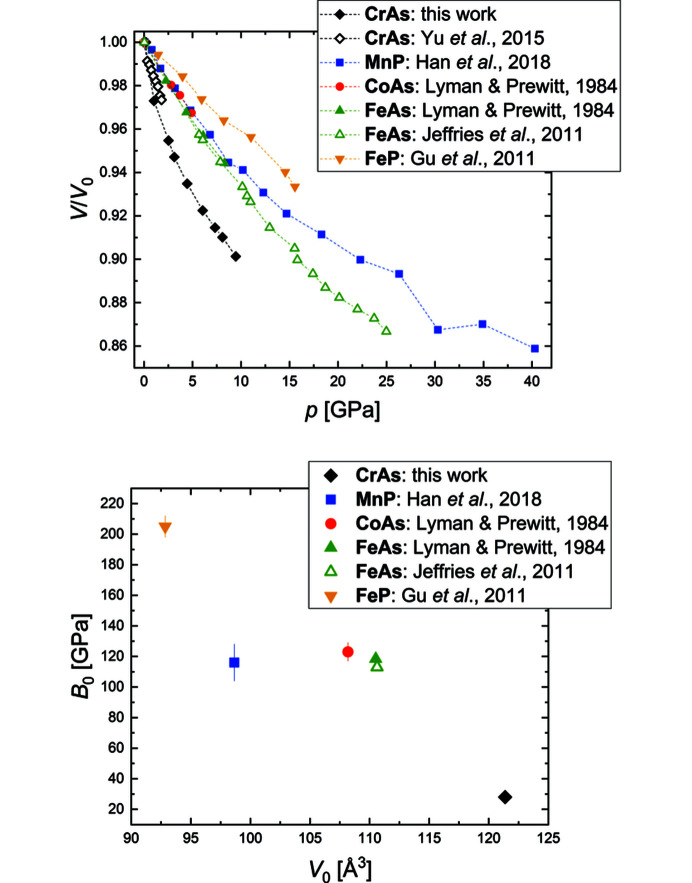
(Top) The unit-cell volume normalized to the value at ambient pressure as function of pressure for various compounds of the MnP-type structure; (bottom) the bulk modulus of those compounds as function of the unit-cell volume at ambient pressure. The dashed lines are guides for the eye.

The pressure step used in this work does not allow a detailed study of the anomalies in the pressure dependence of unit-cell parameters and unit-cell volume below 1 GPa as observed by Yu *et al.* (2015 ▸) in their powder diffraction data. Nevertheless, we confirm that there is no symmetry change in CrAs upon compression to 9.5 GPa at room temperature based on our single-crystal data. Fitting the pressure-dependent unit-cell volumes from the synchrotron X-ray data with a third-order Birch–Murnaghan equation of state (Birch, 1947 ▸) shows that the whole high-pressure range can be fitted with one equation of state (Fig. 4 ▸). The unit-cell parameters and unit-cell volume at ambient pressure and room temperature are *a* = 5.647 (3) Å, *b* = 3.467 (2) Å, *c* = 6.200 (3) Å and *V* = 121.4 (1) Å^3^. The fitted unit-cell volume at zero pressure, the bulk modulus and the first derivative of the bulk modulus are *V*_0_ = 121.3 (2) Å^3^, *B*_0_ = 28 (4) GPa and *B*_0_′ = 36 (5), respectively. Compared to other MnP-type compounds [*e.g.* MnP: *B*_0_ = 116 (12) GPa, *B*_0_′ = 4.2 (8) (Han *et al.*, 2018 ▸), FeP: *B*_0_ = 205 (7) GPa, *B*_0_′ = 4 (Gu *et al.*, 2011 ▸), CoAs: *B*_0_ = 123 (6) GPa, *B*_0_′ = 8.8 (33) (Lyman & Prewitt, 1984 ▸), FeAs: *B*_0_ = 113.5 GPa, *B*_0_′ = 5.7 (Jeffries *et al.*, 2011 ▸)], the bulk modulus of CrAs is very low and its first derivative very high. The low bulk modulus reflects the significantly stronger compressibility of CrAs compared to the other compounds (Fig. 5 ▸, top). The large *B*_0_′ might be due to large, initial changes in the interatomic distances and could also possibly be related to a phase transition that is, however, not evident in the unit-cell volume. While the underlying reasons for the respective behaviors on compression are not elucidated, the bulk moduli are negatively correlated with the unit-cell volume (Fig. 5 ▸, bottom), with CrAs having the largest unit cell and the lowest bulk modulus, which indicates that this might play a main role in the compressibility.

### 3.4. Refinements and twinning   

**Figure 6:**
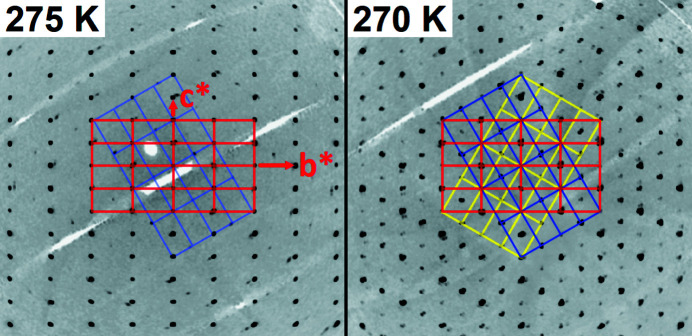
Reconstructions of the reciprocal space at 275 K (left) and 270 K (right) based on the synchrotron X-ray measurements. The main twin orientations used for the respective indexing are indicated.

As mentioned before, CrAs undergoes a phase transition from the parent NiAs-type structure to the MnP-type structure at high temperatures which is coupled to the formation of twin domains with the threefold rotational axis being the twinning element. The passing of this transition temperature in the growth process of CrAs explains the frequent occurrence of twin domains in these crystals. The single crystals used were selected to be largely composed of one single domain, but the presence of initial low-volume twin domains can be seen in the reciprocal space reconstructions of the measurements as a splitting of a part of the reflections and of additional reflections that are not indexed by the primary twin (Fig. 6 ▸, left); 97.5% of the reflections can be indexed by the primary twin, the second twin component indexes 2.2% of the previously unindexed reflections.

Below the transition temperature *T*_N_, the reciprocal space reconstruction shows a significant change in the domain structure of the CrAs crystal (Fig. 6 ▸, right). The primary twin now indexes only 75.8% of the reflections, and the second and third twin components index additionally 12.2% and 11.1%, respectively. With a further decrease in temperature, the domain structure does not substantially change. For the refinements, only the integrated data based on the reflections that can be indexed by the prime twin component (which shows some overlap with the other two twin components) were used in all cases.

In the high-temperature phase transition, the twinning is coupled to the lowering of the symmetry from *P*6_3_/*mmc* to *Pnma*, with the lost threefold rotational axis along the orthorhombic *a* axis acting as the twinning element. The same twinning element is observed in the twinning at *T*_N_, although the transition is isosymmetrical and this threefold rotational axis does not play a direct role in the transition. We believe that the reason for this twinning is related to the change in the *c*/*b* ratio mentioned before (Fig. 3 ▸). During the change from *c*/*b* > 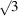 to *c*/*b* < 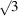 , the structure has to briefly assume *c/b* = 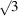 , in which the unit-cell parameters correspond to the orthohexagonal setting, while the atoms are slightly shifted from their ideal orthohexagonal positions. In this pseudohexagonal setting, a pseudo-threefold axis is present in the structure, which could act as the twinning element during the subsequent loss of the pseudosymmetry, leading to the formation of the observed twin domains below the transition. The larger part of the volume keeps the orientation of the primary twin, while the two twin orientations with ±120° are present with lower but approximately the same volume fractions.

This formation of twin domains during the phase transition is furthermore coupled to increased displacement parameters of the atoms (Fig. S1). In addition to the standard refinements with harmonic atomic displacement parameters (ADPs), refinements with anharmonic ADPs were carried out, as this was suggested by a visual inspection of the *F*_obs_ density maps. The resulting parameters show consistently that around the transition temperature the displacement parameters of the atoms show a significant anharmonicity, indicating that around and during the phase transition, the atoms show increased and anharmonic movement around their mean positions or that there is an increased degree of static disorder of the atoms which are slightly moved away from their highly symmetrical special positions.

The change in the twin domain structure during the phase transition at *T*_N_ does naturally mean a significant change in the microstructure of the CrAs single crystals.^2^ However, the change in the microstructure seems to depend on the temperature rate with which the phase transition is crossed, as the change in the twin domain structure was observed in our synchrotron measurements, but not in the laboratory measurements.^2^

In contrast to the temperature-dependent behavior, the application of pressure does not induce such a change in the microstructure, as the behavior of the unit-cell parameters at high pressures is different: the ideal *c*/*b* ratio of the pseudohexagonal setting is not assumed and no twinning is observed.

The observed change in the twin domain structure has not been reported for any of the other compounds exhibiting an isosymmetrical phase transition between MnP-type structures. As the *c*/*b* = 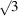 setting is not crossed during those transitions, a twinning phenomenon analogous to the one described for CrAs is also not expected. Hence, the transition in CrAs including this twinning seems to originate from a coincidence based on the specific unit-cell parameters, and is thus not characteristic for the group of MnP-type structure transition-metal pnictides as a whole.

### 3.5. Interatomic distances   

**Figure 7:**
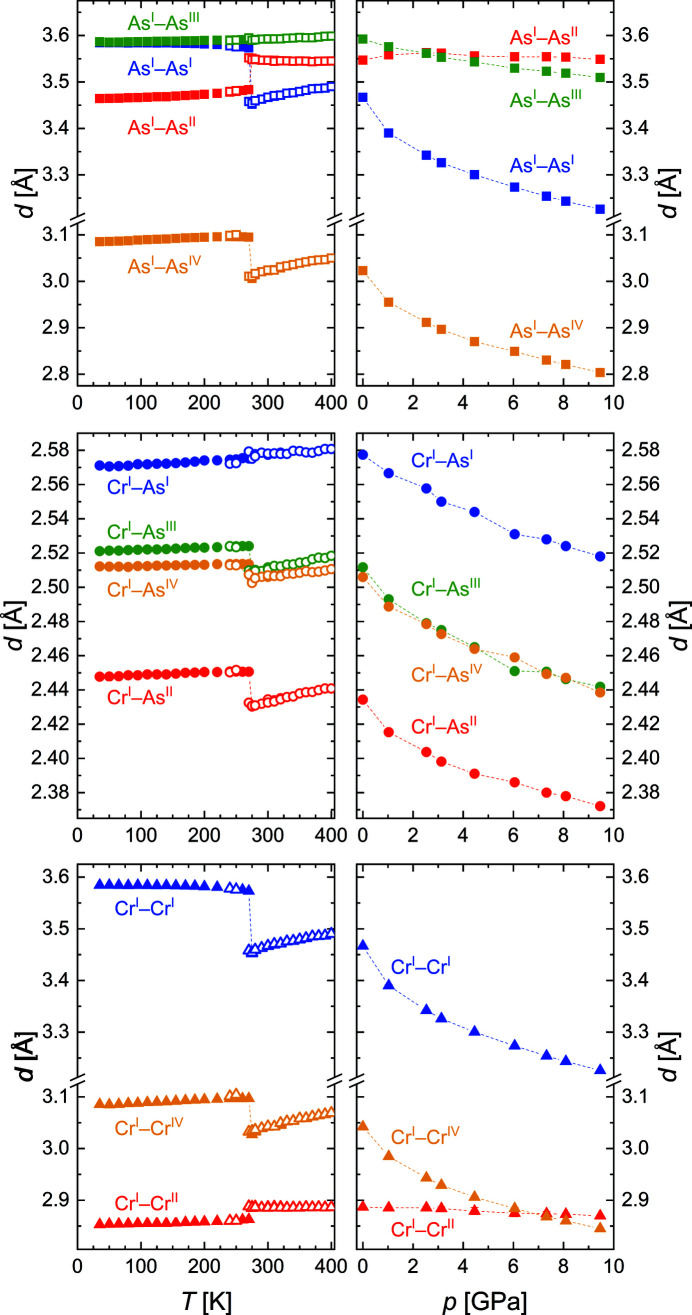
Interatomic As–As (top), Cr–As (middle) and Cr–Cr (bottom) distances as a function of temperature (left) and pressure (right). Data from synchrotron and laboratory X-ray measurements are indicated by filled and open symbols, respectively. The dashed lines are guides for the eye.

The majority of the interatomic distances show the following general behavior. Above the phase transition, they increase with increasing temperature (Fig. 7 ▸). At the transition temperature *T*_N_, they show an abrupt increase, and below the transition they decrease with temperature. In all three regimes, however, exceptions are observed for specific distances.

The most interesting distances in the CrAs structure are the Cr–Cr distances (see also Fig. S2), as the Cr atoms carry the magnetic moment and they and their interactions are of particular importance for the magnetic and eventually superconducting properties. Above the phase transition, the distances Cr^I^–Cr^I^ and Cr^I^–Cr^IV^ follow the general trend and increase with temperature. However Cr^I^—Cr^II^, the strongest covalent Cr—Cr bond and strongest homoatomic interaction, is remarkable as it shows anomalous behavior in that it stays constant above *T*_N_. At the phase transition, the same distinction between the three Cr–Cr distances is seen. Here, Cr^I^–Cr^I^ and Cr^I^–Cr^IV^ increase (upon cooling) due to the phase transition, while Cr^I^–Cr^II^, the shortest Cr–Cr distance, decreases even further. Cr^I^–Cr^I^ directly determines the length of the *b* axis of the unit cell, so that its increase is equivalent to the observed discontinuity in the temperature dependence of the *b* unit-cell parameter. Cr^I^–Cr^IV^, lying almost in the *bc* plane, increases as well. The decrease in Cr^I^–Cr^II^, which is oriented approximately along the *a* axis, leads to the abrupt shortening of this axis. Besides this decrease in the distance Cr^I^–Cr^II^, the transition is also accompanied by a slight straightening of the Cr^I^–Cr^II^–Cr^I^ chains (Fig. S3) and the ∠Cr^I^–Cr^II^–Cr^I^ angle gets closer to the ideal value of 180°. Cr^I^–Cr^II^ thus shows two distinctions: decreasing at *T*_N_ and staying constant above *T*_N_. Below the transition, Cr^I^–Cr^II^ and Cr^I^–Cr^IV^ decrease with temperature, while Cr^I^–Cr^I^ increases and is eventually constant below about 200 K.

**Figure 8:**
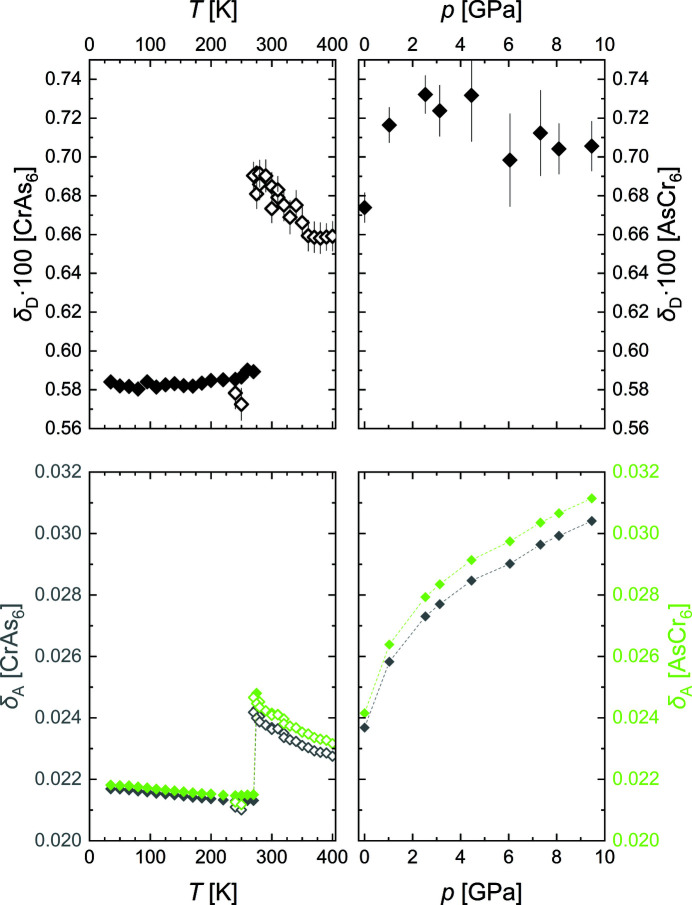
Distance distortion (top) and angular distortion (bottom) of the [CrAs_6_] octahedra and the [AsCr_6_] prisms as a function of temperature (left) and pressure (right). The dashed lines are guides for the eye.

The Cr–As distances, which determine the coordination polyhedra, all increase above *T*_N_ following the general trend. During the phase transition and in contrast to the other Cr–As distances, the longest Cr–As distance, Cr^I^–As^I^, decreases. From this, we infer that both the octahedra and the prisms become more regular in the low-temperature phase. This is also seen in the distance distortion δ_D_ and the angular distortion δ_A_ – calculated as the deviation of the experimental values for the distances and angles, respectively, from the restrictions imposed by the reference shapes (see supporting information more details) – which decrease during the phase transition (Fig. 8 ▸). Below the transition and with further decreasing temperature, the Cr–As distances again all follow the general trend and the distortion of the polyhedra slightly increases again.

The calculated As–As distances show anomalous behavior in the different regimes; however, they reflect the changes in distortion of the coordination polyhedra and are not associated with homoatomic interactions, and one can assume that they follow from the bonding pattern of As with Cr atoms.

In CrAs on compression, all Cr–As and Cr–Cr distances decrease with the exception of Cr^I^–Cr^II^ (Fig. 7 ▸), which is almost constant up to 9.5 GPa. This leads to an increasing distortion of the [CrAs_6_] octahedra and [AsCr_6_] prisms as well as the [CrAs_6_] columns, seen in the pressure dependence of the distortions (Fig. 8 ▸) and the ∠Cr^I^–Cr^II^–Cr^I^ angle (Fig. S3).

## 4. Discussion   

**Figure 9:**
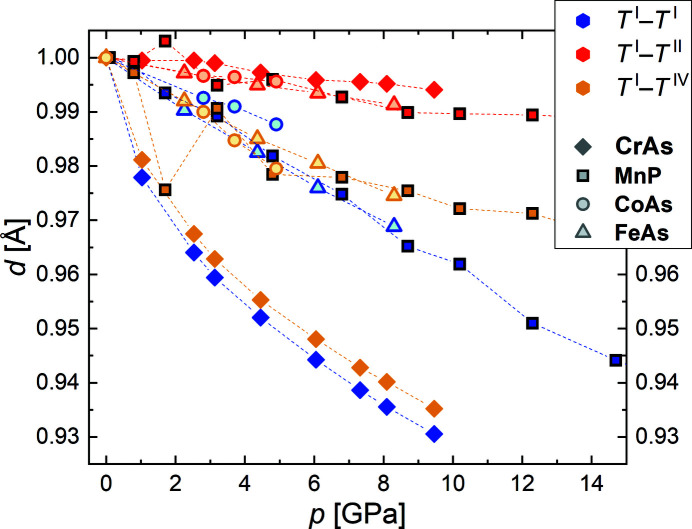
The behavior of the interatomic *T*–*T* distances in transition-metal pnictides as function of pressure. The distance designation follows the one used for CrAs in the present work. The data for MnP are taken from Han *et al.* (2018 ▸) and data for CoAs and FeAs are taken from Lyman & Prewitt (1984 ▸). The dashed lines are guides for the eye.

The measured Cr–Cr distances are in very good agreement with the data reported by Shen *et al.* (2016 ▸) (Fig. S4) and also in good agreement with those reported by Selte *et al.* (1971 ▸). Furthermore, the data measured by Shen *et al.* show that above the phase transition the pressure behavior of Cr^I^–Cr^II^ and Cr^I^–Cr^IV^ does not depend significantly on the temperature; in particular, the results confirm the Cr^I^–Cr^II^ distance remains nearly constant in the paramagnetic phase, both as a function of temperature and of high pressure, and probably even when temperature and pressure are varied simultaneously. Hence, it can be assumed that the present results are likely to be more generally valid. With regard to the pressure behavior of CrAs, a comparison of its structural behavior with related transition-metal pnictides (TPn) with the MnP-type structure shows that at least the behavior of the relevant interatomic *T*–*T* distances on compression are similar (Fig. 9 ▸): *T* ^I^–*T* ^II^ is least affected by the pressure and changes relatively little, while *T* ^I^–*T* ^I^ and *T* ^I^–*T* ^IV^ are affected much more and in similar ways. This tendency is observed in CrAs, MnP, CoAs and FeAs, though the contrast in behavior between *T* ^I^–*T* ^II^ and *T* ^I^–*T* ^I^/*T* ^I^–*T* ^IV^ is most pronounced in CrAs. The lack of temperature-dependent data for the TPn compounds, however, currently prevents a comparison of the *T*–*T* behavior with temperature, and in particular a more comprehensive study of the *T* ^I^–*T* ^II^ distance and its relation to other properties. Nevertheless, the structural similarities observed so far indicate that the present results for CrAs might also be of importance for the other TPn compounds.

In CrAs, above *T*_N_, the Cr^I^–Cr^I^, Cr^I^–Cr^IV^ and Cr–As distances increase following the expected thermal expansion. The Cr^I^–Cr^II^ distance, however, shows in general a significantly different behavior above *T*_N_ in the paramagnetic state. It stays constant with increasing temperature up to 400 K and also remains remarkably constant at pressures up to 9.5 GPa.

At *T*_N_, the observed changes in the structure during the phase transition agree with the literature and can be explained using the electronic model proposed by Zavadskii & Sibarova (1980 ▸). This model can also be taken to explain the behavior of the Cr–Cr distances at lower temperatures. For Cr^I^–Cr^I^ – oriented along the *b* axis, but too far away for an attractive interaction – the partial filling of the 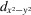 orbital in this direction [with *x*∥*b* and *y*∥*c*, following the notation used by Boller & Kallel (1971 ▸)] during the phase transition leads to the electrostatic repulsion responsible for the abrupt increase in this distance.

Below *T*_N_, the distance increases further and stays constant below ∼200 K. With decreasing temperature, the energy of the 3*d* band is further lowered and the orbitals show increased occupation by electrons. This in turn leads presumably to an increased electrostatic repulsion and the continued increase in distance. The more the temperature decreases below the transition temperature, the less the electron occupation changes, and eventually a constant distance is reached. Furthermore, the change in Cr^I^–Cr^I^ at the transition can be considered as the main reason for the accompanying change in the distance Cr^I^–Cr^IV^. This distance lies approximately in the *bc* plane and is directly coupled to the length of the *b* unit-cell parameter; the increase in the Cr^I^–Cr^I^ distance and the associated increase in the *b* unit-cell parameter might force the increase in the Cr^I^–Cr^IV^ distances as a side effect. As noted by Boller & Kallel (1971 ▸), it is this increase of the Cr^I^–Cr^IV^ distance close to the Cr–Cr Mott critical distance of 3.18 Å for the transition from itinerant to localized electrons that is responsible for the localized magnetic moment at low temperatures. According to this, the magnetic transition follows the electronic and structural transition, but no indication of a separation of the two transitions has been observed so far (Matsuda *et al.*, 2018 ▸). Above *T*_N_, those two distances increase following the expected thermal expansion. The application of pressure at room temperature in the paramagnetic state above the phase transition leads to a decrease in these two distances with increasing pressure as the respective atoms are pushed closer, with no hints towards obvious changes in the electronic interactions between Cr^I^–Cr^I^ and Cr^I^–Cr^IV^.

In contrast, Cr^I^–Cr^II^ shows a completely different behavior during the phase transition and in the paramagnetic state with respect to its temperature and pressure dependence. As the shortest Cr–Cr distance within the structure it is expected to have the strongest Cr–Cr interactions with a strong covalent bond (Zavadskii & Sibarova, 1980 ▸), which should result in mostly itinerant electrons that do not significantly contribute to the ordered magnetic moment. The behavior of Cr^I^–Cr^II^ during the phase transition can also be explained within the Zavadskii & Sibarova model. Below the phase transition, electrons occupy the 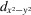 orbital along the *b* direction responsible for the electrostatic repulsion. At *T*_N_, thermal transfer from this orbital to an antibonding 3*d* orbital oriented along Cr^I^–Cr^II^ is possible as the energy gap is sufficiently reduced. The increased occupancy of this antibonding orbital weakens the interatomic bond, resulting in an increase of this distance. While the differences in behavior going through the phase transition can thus be explained within this model, it does not explain the differences in behavior in the paramagnetic state mentioned earlier, where Cr^I^–Cr^II^ is distinguished by being nearly unaffected by temperature and pressure. The possible significance of this particular nearest-neighbor Cr distance has been noted by Shen *et al.* (2016 ▸), whose measurements in the antiferromagnetic phase near the superconducting phase region indicate that the spins of these Cr atoms rotate towards an almost antiparallel order. This might suggest that the superconductivity is mediated by antiferromagnetic fluctuations involving these two Cr atoms, where the long-range helical magnetic order is suppressed by pressure and short-range antiferromagnetic fluctuations along the *a* axis might be responsible for a superconducting electron coupling. The competition of the mutually exclusive long-range order responsible for the magnetism and the short-range fluctuations responsible for the superconductivity is evident in the evolution of the magnetic/superconducting volume fractions with pressure (Keller *et al.*, 2015 ▸; Khasanov *et al.*, 2015 ▸; Shen *et al.*, 2016 ▸). Hence, as the nearest-neighbor Cr^I^–Cr^II^ distance is assumed to govern the interaction between the spins, it might play an important role in the emergence of the superconductivity. However, a direct determination of the role of the crystal structure of CrAs and in particular of the Cr^I^–Cr^II^ distance in the mechanism of superconductivity would require detailed investigations of the crystal structure in and near the superconducting region.

## 5. Conclusion   

Two distinct anomalies have been observed for CrAs in dependence on temperature and on pressure. First, the isosymmetrical magnetostructural phase transition can induce a change in the microstructure, where twinning is caused by the crossing of the pseudohexagonal setting. Such a behavior has, to the best of our knowledge, not yet been reported for any of the transition-metal arsenides. Second, within the crystal structure of CrAs, the shortest Cr–Cr distance, Cr^I^–Cr^II^, exhibits remarkable behavior in that it stays nearly constant in the paramagnetic region and is not significantly affected by temperature and pressure. As this particular distance has been noted before to potentially play an important role in the mechanism of the superconductivity in CrAs, this highlights the importance of further dedicated studies of the relationship of the crystal structure and the superconductivity.

## 6. Related literature   

The following references are cited in the supporting information: Baur (1974 ▸); Brown & Shannon (1973 ▸); Guionneau *et al.* (2002 ▸); Robinson *et al.* (1971 ▸); Tarakina *et al.* (2003 ▸); Wang & Liebau (2007 ▸); Zhang *et al.* (2019 ▸).

## Supplementary Material

Crystal structure: contains datablock(s) global, Synchrotron-275K, Synchrotron-270K, Synchrotron-260K, Synchrotron-250K, Synchrotron-240K, Synchrotron-220K, Synchrotron-200K, Synchrotron-185K, Synchrotron-170K, Synchrotron-155K, Synchrotron-140K, Synchrotron-125K, Synchrotron-110K, Synchrotron-95K, Synchrotron-80K, Synchrotron-65K, Synchrotron-50K, Synchrotron-35K, Synchrotron-1.03GPa, Synchrotron-2.53GPa, Synchrotron-3.13GPa, Synchrotron-4.45GPa, Synchrotron-6.05GPa, Synchrotron-7.32GPa, Synchrotron-8.09GPa, Synchrotron-9.46GPa, Lab-400K, Lab-390K, Lab-380K, Lab-370K, Lab-360K, Lab-350K, Lab-340K, Lab-330K, Lab-320K_02, Lab-320K_01, Lab-310K_02, Lab-310K_01, Lab-300K_02, Lab-300K_01, Lab-290K, Lab-280K_02, Lab-280K_01, Lab-275K, Lab-270K, Lab-250K, Lab-240K. DOI: 10.1107/S2052520621005655/xk5084sup1.cif

Structure factors: contains datablock(s) Synchrotron-275K. DOI: 10.1107/S2052520621005655/xk5084Synchrotron-275Ksup2.hkl

Structure factors: contains datablock(s) Synchrotron-270K. DOI: 10.1107/S2052520621005655/xk5084Synchrotron-270Ksup3.hkl

Structure factors: contains datablock(s) Synchrotron-260K. DOI: 10.1107/S2052520621005655/xk5084Synchrotron-260Ksup4.hkl

Structure factors: contains datablock(s) Synchrotron-250K. DOI: 10.1107/S2052520621005655/xk5084Synchrotron-250Ksup5.hkl

Structure factors: contains datablock(s) Synchrotron-40K. DOI: 10.1107/S2052520621005655/xk5084Synchrotron-240Ksup6.hkl

Structure factors: contains datablock(s) Synchrotron-220K. DOI: 10.1107/S2052520621005655/xk5084Synchrotron-220Ksup7.hkl

Structure factors: contains datablock(s) Synchrotron-200K. DOI: 10.1107/S2052520621005655/xk5084Synchrotron-200Ksup8.hkl

Structure factors: contains datablock(s) Synchrotron-185K. DOI: 10.1107/S2052520621005655/xk5084Synchrotron-185Ksup9.hkl

Structure factors: contains datablock(s) Synchrotron-170K. DOI: 10.1107/S2052520621005655/xk5084Synchrotron-170Ksup10.hkl

Structure factors: contains datablock(s) Synchrotron-155K. DOI: 10.1107/S2052520621005655/xk5084Synchrotron-155Ksup11.hkl

Structure factors: contains datablock(s) Synchrotron-140K. DOI: 10.1107/S2052520621005655/xk5084Synchrotron-140Ksup12.hkl

Structure factors: contains datablock(s) Synchrotron-125K. DOI: 10.1107/S2052520621005655/xk5084Synchrotron-125Ksup13.hkl

Structure factors: contains datablock(s) Synchrotron-110K. DOI: 10.1107/S2052520621005655/xk5084Synchrotron-110Ksup14.hkl

Structure factors: contains datablock(s) Synchrotron-95K. DOI: 10.1107/S2052520621005655/xk5084Synchrotron-95Ksup15.hkl

Structure factors: contains datablock(s) Synchrotron-80K. DOI: 10.1107/S2052520621005655/xk5084Synchrotron-80Ksup16.hkl

Structure factors: contains datablock(s) Synchrotron-65K. DOI: 10.1107/S2052520621005655/xk5084Synchrotron-65Ksup17.hkl

Structure factors: contains datablock(s) Synchrotron-50K. DOI: 10.1107/S2052520621005655/xk5084Synchrotron-50Ksup18.hkl

Structure factors: contains datablock(s) Synchrotron-35K. DOI: 10.1107/S2052520621005655/xk5084Synchrotron-35Ksup19.hkl

Structure factors: contains datablock(s) Synchrotron-1.03GPa. DOI: 10.1107/S2052520621005655/xk5084Synchrotron-1.03GPasup20.hkl

Structure factors: contains datablock(s) Synchrotron-2.53GPa. DOI: 10.1107/S2052520621005655/xk5084Synchrotron-2.53GPasup21.hkl

Structure factors: contains datablock(s) Synchrotron-3.13GPa. DOI: 10.1107/S2052520621005655/xk5084Synchrotron-3.13GPasup22.hkl

Structure factors: contains datablock(s) Synchrotron-4.45GPa. DOI: 10.1107/S2052520621005655/xk5084Synchrotron-4.45GPasup23.hkl

Structure factors: contains datablock(s) Synchrotron-6.05GPa. DOI: 10.1107/S2052520621005655/xk5084Synchrotron-6.05GPasup24.hkl

Structure factors: contains datablock(s) Synchrotron-7.32GPa. DOI: 10.1107/S2052520621005655/xk5084Synchrotron-7.32GPasup25.hkl

Structure factors: contains datablock(s) Synchrotron-8.09GPa. DOI: 10.1107/S2052520621005655/xk5084Synchrotron-8.09GPasup26.hkl

Structure factors: contains datablock(s) Synchrotron-9.46GPa. DOI: 10.1107/S2052520621005655/xk5084Synchrotron-9.46GPasup27.hkl

Structure factors: contains datablock(s) Lab-400K. DOI: 10.1107/S2052520621005655/xk5084Lab-400Ksup28.hkl

Structure factors: contains datablock(s) Lab-390K. DOI: 10.1107/S2052520621005655/xk5084Lab-390Ksup29.hkl

Structure factors: contains datablock(s) Lab-380K. DOI: 10.1107/S2052520621005655/xk5084Lab-380Ksup30.hkl

Structure factors: contains datablock(s) Lab-370K. DOI: 10.1107/S2052520621005655/xk5084Lab-370Ksup31.hkl

Structure factors: contains datablock(s) Lab-360K. DOI: 10.1107/S2052520621005655/xk5084Lab-360Ksup32.hkl

Structure factors: contains datablock(s) Lab-350K. DOI: 10.1107/S2052520621005655/xk5084Lab-350Ksup33.hkl

Structure factors: contains datablock(s) Lab-340K. DOI: 10.1107/S2052520621005655/xk5084Lab-340Ksup34.hkl

Structure factors: contains datablock(s) Lab-330K. DOI: 10.1107/S2052520621005655/xk5084Lab-330Ksup35.hkl

Structure factors: contains datablock(s) Lab-320K_02. DOI: 10.1107/S2052520621005655/xk5084Lab-320K_02sup36.hkl

Structure factors: contains datablock(s) Lab-320K_01. DOI: 10.1107/S2052520621005655/xk5084Lab-320K_01sup37.hkl

Structure factors: contains datablock(s) Lab-310K_02. DOI: 10.1107/S2052520621005655/xk5084Lab-310K_02sup38.hkl

Structure factors: contains datablock(s) Lab-310K_01. DOI: 10.1107/S2052520621005655/xk5084Lab-310K_01sup39.hkl

Structure factors: contains datablock(s) Lab-300K_02. DOI: 10.1107/S2052520621005655/xk5084Lab-300K_02sup40.hkl

Structure factors: contains datablock(s) Lab-300K_01. DOI: 10.1107/S2052520621005655/xk5084Lab-300K_01sup41.hkl

Structure factors: contains datablock(s) Lab-290K. DOI: 10.1107/S2052520621005655/xk5084Lab-290Ksup42.hkl

Structure factors: contains datablock(s) Lab-280K_02. DOI: 10.1107/S2052520621005655/xk5084Lab-280K_02sup43.hkl

Structure factors: contains datablock(s) Lab-280K_01. DOI: 10.1107/S2052520621005655/xk5084Lab-280K_01sup44.hkl

Structure factors: contains datablock(s) Lab-275K. DOI: 10.1107/S2052520621005655/xk5084Lab-275Ksup45.hkl

Structure factors: contains datablock(s) Lab-240K. DOI: 10.1107/S2052520621005655/xk5084Lab-240Ksup46.hkl

Structure factors: contains datablock(s) Lab-250K. DOI: 10.1107/S2052520621005655/xk5084Lab-250Ksup47.hkl

Structure factors: contains datablock(s) Lab-270K. DOI: 10.1107/S2052520621005655/xk5084Lab-270Ksup48.hkl

Tables S1-S12 , Appendix A and Figs. S1-S4. DOI: 10.1107/S2052520621005655/xk5084sup49.pdf

CCDC references: 2087357, 2087358, 2087359, 2087360, 2087361, 2087362, 2087363, 2087364, 2087365, 2087366, 2087367, 2087368, 2087369, 2087370, 2087371, 2087372, 2087373, 2087374, 2087375, 2087376, 2087377, 2087378, 2087379, 2087380, 2087381, 2087382, 2087383, 2087384, 2087385, 2087386, 2087387, 2087388, 2087389, 2087390, 2087391, 2087392, 2087393, 2087394, 2087395, 2087396, 2087397, 2087398, 2087399, 2087400, 2087401, 2087402, 2087403

## Figures and Tables

**Table 1:** Overview of the structural transitions of transition-metal pnictide (TPn) compounds exhibiting the MnP-type structure The reference shorthands are explained below the table. FM indicates ferromagnetic order; ∥*c* indicates ferromagnetically ordered spins oriented along the *c* axis; HM indicates helimagnetic order; ↑*c* indicates helimagnetic propagation vector along the *c* axis; FS indicates full structure; UC indicates unit-cell parameters; TE indicates thermal expansion. Reference 9 is in parentheses to indicte it is relevant but does not directly give actual data.

		Transition						
TPn	Magnetic order	Order	Structure (cooling)	Temperature	Hysteresis	Pressure	Available structural data	Superconductivity
CrP	No^[1]^	Second^[1]^	(NiAs→MnP)^[1]^	[>1350 (50) K]^[1]^	–	Ambient	FS: 1.2 K, 4.2 K, 293 K^[1]^ 17 K, 81 K, 293 K^[2]^ 293 K^[3–6]^	–
							UC: 4–1200 K^[7]^	
MnP	FM (∥*c*)^[8]^	Second^[9]^	MnP→MnP^[10]^	291.5 (2) K^[11]^	–	Ambient (suppressed by *p*)	FS: 10 K, 60 K, 293 K^[10]^ 293^[3,13,14]^ 0–40 GPa^[15]^	Yes (*p*-induced) *p*_c_ ≃ 7.8 GPa, *T*_c_ ≤ 1 K^[19]^
	HM (↑*c*)^[8]^ (Complex magnetic *p*-*T*-*H*-diagram)	First^[9]^	MnP→MnP*^[10]^ *No crossing of *c*/*b* = {\surd 3}	47 K	0.24 K^[9]^	Ambient (suppressed at *p* ≃ 1.4 GPa)	UC: 300–1300 K^[16]^0.5–7 GPa^[17]^ TE: 4–80 K^[18,(9)]^	
FeP	HM (↑*c*)^[20]^	(Second)^[21]^	MnP→MnP^[21]^	125 (1) K^[20]^	–	Ambient	FS: 293 K^[3,20,22]^ 30–300 K^[21]^	*–*
							UC: 30–1300 K^[16]^ 0–16 GPa^[23]^	
							TE: 100–293 K^[24]^	
CoP	No^[16,25]^						FS: 293 K^[3]^	–
							UC: 300–1300 K^[7]^	
VAs	No^[26]^						FS: 293 K^[26,27]^	–
							UC: 4–1200 K^[7]^	
CrAs	HM (↑*c*)^[28]^	Second^[7]^	NiAs→MnP^[7]^	1170 (20) K^[7]^	–	Ambient	FS: 90 K, 285 K^[29]^ 80 K, 285 K^[32]^ 90 K, 295 K^[33]^ 1.5 K, 80 K, 300 K^[34]^ (1.5–290 K) @ 0–0.94 GPa^[35]^	Yes (*p*-induced) *p*_c_ ≃ 0.4 GPa, *T*_c_ ≤ 2.2 K^[37]^
		First^[29]^	MnP→MnP^[30]^	267 K^[31]^	7 K^[31]^	Ambient (suppressed at *p* ≃ 0.8 GPa)	UC: 90–320 K^[30]^ 90–300 K^[36]^ 100–1300 K^[7]^	
MnAs	FM^[38]^ (Complex magnetic *p*-*T*-*H*-diagram)	Second^[38]^	NiAs→MnP^[38]^	394 K^[38]^	–	Ambient (suppressed by *p*)	FS: 293 K^[39, 40]^ 328 K^[41]^ 284 K, 317 K^[42]^ 4.2 K, 298 K^[43]^ (15 K, 295 K) @ 3.8 GPa^[44]^	–
		First^[38]^	MnP→NiAs^[38]^	306 K^[38]^	11 K^[38]^	Ambient (suppressed by *p*)	UC: 100–500 K^[45]^	
FeAs	HM (↑*c*)^[46]^	(Second)^[46]^	MnP→MnP^[46]^	77 (1) K^[46]^	–	Ambient	FS: 12–293 K^[46]^ 293 K^[47]^ 0–8.3 GPa^[48]^	–
							UC: 12–1325 K^[46]^	
CoAs	No^[49]^	Second^[7]^	NiAs→MnP^[50]^	1225 K^[50]^	–	Ambient	FS: 293 K^[49]^ 0–4.9 GPa^[48]^	–
							UC: 4–1200 K^[7]^	

References: [1] Selte *et al.* (1975 ▸); [2] Selte *et al.* (1972*b* ▸); [3] Rundqvist & Nawapong (1965 ▸); [4] Schönberg (1954 ▸); [5] Boller & Nowotny (1965 ▸); [6] Nowotny & Henglein (1938 ▸); [7] Selte & Kjekshus (1973*a* ▸); [8] Wang *et al.* (2016 ▸); [9] Shapira *et al.* (1981 ▸); [10] Fjellvåg & Kjekshus (1984 ▸); [11] Huber & Ridgley (1964 ▸); [12] Komatsubara *et al.* (1970 ▸); [13] Fjellvåg & Kjekshus (1986 ▸); [14] Zhigadlo *et al.* (2017 ▸); [15] Han *et al.* (2018 ▸); [16] Selte *et al.* (1978 ▸); [17] Yano *et al.* (2018 ▸); [18] Okamoto *et al.* (1968 ▸); [19] Cheng *et al.* (2015 ▸); [20] Felcher *et al.* (1971 ▸); [21] Chernyavskii *et al.* (2020 ▸); [22] Selte & Kjekshus (1972 ▸); [23] Gu *et al.* (2011 ▸); [24] Westerstrandh *et al.* (1977 ▸); [25] Motizuki *et al.* (2009 ▸); [26] Selte *et al.* (1972*c* ▸); [27] Bachmayer & Nowotny (1955 ▸); [28] Watanabe *et al.* (1969 ▸); [29] Boller & Kallel (1971 ▸); [30] Suzuki & Ido (1991 ▸); [31] Wu *et al.* (2010 ▸); [32] Selte *et al.* (1971 ▸); [33] Sen *et al.* (2019 ▸); [34] Keller *et al.* (2015 ▸); [35] Shen *et al.* (2016 ▸); [36] Kanaya *et al.* (2004 ▸); [37] Wu *et al.* (2014 ▸); [38] Zięba *et al.* (1982 ▸); [39] Nowotny *et al.* (1951 ▸); [40] Yuzuri & Yamada (1960 ▸); [41] Wilson & Kasper (1964 ▸); [42] Suzuki (1978 ▸); [43] Zięba *et al.* (1978 ▸); [44] Glazkov *et al.* (2003 ▸); [45] Suzuki & Ido (1982 ▸); [46] Selte *et al.* (1972*a* ▸); [47] Selte & Kjekshus (1969 ▸); [48] Lyman & Prewitt (1984 ▸); [49] Selte & Kjekshus (1971 ▸); [50] Heyding & Calvert (1957 ▸).

## Footnotes

1 These transitions are commonly denoted as isostructural in the literature. However, we prefer the term isosymmetrical, as the symmetry of both phases is identical, while there are significant changes in the structure.

2 This change in the microstructure might affect the emergence of superconductivity in CrAs, which is very sensitive to the residual resistivity and thus to local disorder and strains (Matsuda *et al.*, 2018 ▸).
