# Supplementary material for: Comparison of the temperature- and pressure-dependent behavior of the crystal structure of CrAs
Source: Acta Crystallogr B Struct Sci Cryst Eng Mater. 2021 Jul 23;77(Pt 4):594–604. doi: 10.1107/S2052520621005655 (PMC8337019; doi:10.1107/S2052520621005655)
Supplement: Supplementary file 49 [file b-77-00594-sup49.pdf]

STRUCTURAL SCIENCE
CRYSTAL ENGINEERING
MATERIALS

**Volume 77 (2021)**

**Supporting information for article:**

# Comparison of the temperature- and pressure-dependent behavior of the crystal structure of CrAs

**Andreas Eich, Andrzej Grzechnik, Carsten Paulmann, Thomas Müller, Yixi Su, Thomas Wolf and Karen Friese**

**Table S1** Refinement details of the temperature-dependent synchrotron X-ray measurements on CrAs at ambient pressure. Refinements where not all parameters could be refined are marked: * the extinction factor $G_{iso}$ could not be refined; † only one twin component could be refined; ‡ no twin component could be refined.

| $T$ [K] | No. meas. Refl. (obs/all) | $h_{min} \rightarrow h_{max}$ $k_{min} \rightarrow k_{max}$ $l_{min} \rightarrow l_{max}$ | No. sym. ind. Refl. (obs/all) | $R$(int) (obs/all) | $R$ (obs/all) | $wR$ (obs/all) | No. param. | Space group | $a$ [Å] | $b$ [Å] | $c$ [Å] | $V$ [Å³] |
|---|---|---|---|---|---|---|---|---|---|---|---|---|
| 275 | 13472/15537 | −14 → 16<br>−10 → 10<br>−17 → 18 | 1571/1718 | 7.88/7.88 | 3.13/3.23 | 3.80/3.81 | 14 | *Pnma* | 5.64298(8) | 3.45198(5) | 6.20197(8) | 120.811(3) |
| 270 | 14015/15983 | −14 → 16<br>−10 → 10<br>−17 → 17 | 1646/1793 | 6.81/6.82 | 3.966/3.75 | 4.75/4.76 | 15* | *Pnma* | 5.62063(9) | 3.57308(6) | 6.15072(10) | 123.525(4) |
| 260 | 13936/16041 | −16 → 14<br>−10 → 10<br>−17 → 17 | 1613/1758 | 7.21/7.22 | 3.49/3.58 | 4.39/4.40 | 15* | *Pnma* | 5.61887(10) | 3.57531(6) | 6.14917(11) | 123.532(4) |
| 250 | 13938/15994 | −14 → 16<br>−10 → 10<br>−17 → 17 | 1619/1758 | 7.73/7.74 | 2.81/2.93 | 3.84/3.85 | 16 | *Pnma* | 5.61729(9) | 3.57721(6) | 6.14676(11) | 123.514(3) |
| 240 | 13929/15981 | −14 → 16<br>−10 → 10<br>−17 → 17 | 1622/1757 | 7.13/7.14 | 2.41/2.53 | 3.29/3.31 | 16 | *Pnma* | 5.61599(10) | 3.57836(6) | 6.14550(11) | 123.500(4) |
| 220 | 14049/15996 | −14 → 16<br>−10 → 10<br>−17 → 17 | 1623/1757 | 6.57/6.58 | 2.34/2.46 | 3.17/3.20 | 16 | *Pnma* | 5.61290(11) | 3.58055(7) | 6.14272(12) | 123.452(4) |
| 200 | 13924/15919 | −14 → 16<br>−10 → 10<br>−17 → 17 | 1623/1755 | 7.76/7.76 | 2.56/2.69 | 3.36/3.37 | 16 | *Pnma* | 5.61059(12) | 3.58148(8) | 6.14112(13) | 123.401(5) |
| 185 | 13865/15795 | −14 → 16<br>−10 → 10<br>−17 → 17 | 1629/1756 | 6.84/6.84 | 2.23/2.38 | 2.96/3.02 | 15† | *Pnma* | 5.60945(12) | 3.58259(7) | 6.13862(13) | 123.364(4) |
| 170 | 14009/15871 | −14 → 16<br>−10 → 10<br>−17 → 17 | 1631/1755 | 6.68/6.68 | 2.26/2.37 | 2.94/2.96 | 15† | *Pnma* | 5.60797(12) | 3.58313(8) | 6.13709(13) | 123.319(5) |

**Table S1 (cont.)** Refinement details of the temperature-dependent synchrotron X-ray measurements on CrAs at ambient pressure. Refinements where not all parameters could be refined are marked: * the extinction factor $G_{iso}$ could not be refined; † only one twin component could be refined; ‡ no twin component could be refined.

| $T$ [K] | No. meas. Refl. (obs/all) | $h_{min} \rightarrow h_{max}$<br>$k_{min} \rightarrow k_{max}$<br>$l_{min} \rightarrow l_{max}$ | No. sym. ind. Refl. (obs/all) | $R$(int) (obs/all) | $R$ (obs/all) | $wR$ (obs/all) | No. param. | Space group | $a$ [Å] | $b$ [Å] | $c$ [Å] | $V$ [Å³] |
|---|---|---|---|---|---|---|---|---|---|---|---|---|
| 155 | 13877/15765 | −14 → 16<br>−10 → 10<br>−17 → 17 | 1637/1756 | 7.31/7.32 | 2.31/2.41 | 3.12/3.14 | 15† | *Pnma* | 5.60599(13) | 3.58348(8) | 6.13566(14) | 123.259(5) |
| 140 | 13751/15666 | −16 → 14<br>−10 → 10<br>−17 → 17 | 1625/1756 | 7.65/7.66 | 2.58/2.70 | 3.43/3.44 | 15† | *Pnma* | 5.60404(14) | 3.58394(8) | 6.13451(15) | 123.209(5) |
| 125 | 13839/15652 | −16 → 14<br>−10 → 10<br>−17 → 17 | 1633/1756 | 7.12/7.13 | 2.38/2.50 | 3.21/3.23 | 15† | *Pnma* | 5.60354(14) | 3.58370(8) | 6.13403(15) | 123.180(5) |
| 110 | 14014/15773 | −14 → 16<br>−10 → 10<br>−17 → 17 | 1641/1755 | 6.86/6.87 | 2.20/2.30 | 3.11/3.12 | 15† | *Pnma* | 5.60289(13) | 3.58401(8) | 6.13266(15) | 123.149(5) |
| 95 | 14029/15734 | −14 → 16<br>−10 → 10<br>−17 → 17 | 1646/1755 | 6.87/6.87 | 2.08/2.19 | 2.90/2.92 | 15† | *Pnma* | 5.60187(14) | 3.58416(8) | 6.13183(15) | 123.115(5) |
| 80 | 13424/15253 | −14 → 16<br>−10 → 10<br>−17 → 17 | 1628/1753 | 8.45/8.46 | 2.90/3.01 | 3.98/3.99 | 15† | *Pnma* | 5.60069(11) | 3.58377(7) | 6.13051(12) | 123.049(4) |
| 65 | 14064/15701 | −14 → 16<br>−10 → 10<br>−17 → 17 | 1647/1754 | 6.31/6.32 | 1.90/2.00 | 2.52/2.54 | 15† | *Pnma* | 5.59944(10) | 3.58433(6) | 6.12896(11) | 123.010(4) |
| 50 | 14038/15655 | −14 → 16<br>−10 → 10<br>−17 → 17 | 1650/1753 | 6.61/6.61 | 1.98/2.08 | 2.62/2.64 | 15† | *Pnma* | 5.59880(10) | 3.58443(6) | 6.12843(11) | 122.988(4) |
| 35 | 13035/15114 | −14 → 16<br>−10 → 10<br>−17 → 17 | 1614/1751 | 10.38/10.39 | 4.134.28 | 5.91/5.93 | 15† | *Pnma* | 5.59704(12) | 3.58436(8) | 6.13032(14) | 122.985(5) |

**Table S2** Refinement details of the pressure-dependent synchrotron X-ray measurements on CrAs at room temperature. Refinements where not all parameters could be refined are marked: * the extinction factor $G_{iso}$ could not be refined; † only one twin component could be refined; ‡ no twin component could be refined.

| *p* [GPa] | No. meas. Refl. (obs/all) | $h_{min} \rightarrow h_{max}$ $k_{min} \rightarrow k_{max}$ $l_{min} \rightarrow l_{max}$ | No. sym. ind. Refl. (obs/all) | *R*(int) (obs/all) | *R* (obs/all) | *wR* (obs/all) | No. param. | Space group | *a* [Å] | *b* [Å] | *c* [Å] | *V* [Å³] |
|---|---|---|---|---|---|---|---|---|---|---|---|---|
| 1.03 | 1437/2173 | −9 → 9<br>−4 → 4<br>−10 → 10 | 195/268 | 5.99/6.19 | 3.12/4.36 | 3.55/3.65 | 15† | *Pnma* | 5.6271(5) | 3.3902(10) | 6.1909(7) | 118.10(12) |
| 2.53 | 1367/2192 | −9 → 9<br>−4 → 4<br>−10 → 10 | 185/265 | 8.72/9.03 | 3.54/5.47 | 3.70/3.87 | 14*† | *Pnma* | 5.6147(5) | 3.3418(11) | 6.1760(9) | 115.88(4) |
| 3.13 | 1322/2079 | −9 → 9<br>−5 → 5<br>−10 → 10 | 222/300 | 8.90/9.09 | 4.25/5.47 | 4.59/4.67 | 14‡ | *Pnma* | 5.610(2) | 3.3255(11) | 6.162(2) | 114.96(7) |
| 4.45 | 1421/1991 | −7 → 6<br>−5 → 5<br>−10 → 10 | 202/242 | 14.65/14.79 | 7.56/8.39 | 8.19/8.29 | 14‡ | *Pnma* | 5.593(5) | 3.3003(8) | 6.1478(13) | 113.47(10) |
| 6.05 | 683/1029 | −7 → 7<br>−5 → 5<br>−10 → 10 | 169/207 | 6.64/6.76 | 7.15/7.62 | 8.42/8.43 | 14‡ | *Pnma* | 5.581(3) | 3.2734(6) | 6.1287(10) | 111.97(7) |
| 7.32 | 1722/1984 | −9 → 9<br>−5 → 5<br>−8 → 8 | 226/247 | 8.34/8.35 | 4.20/4.33 | 4.90/4.91 | 15† | *Pnma* | 5.5765(9) | 3.2539(4) | 6.118(2) | 111.01(5) |
| 8.09 | 1050/1742 | −9 → 9<br>−5 → 5<br>−10 → 9 | 212/283 | 7.75/8.03 | 4.73/7.55 | 4.91/5.17 | 14‡ | *Pnma* | 5.5721(12) | 3.2432(7) | 6.1128(14) | 110.47(4) |
| 9.46 | 1678/1967 | −9 → 9<br>−5 → 5<br>−8 → 8 | 210/241 | 7.50/7.52 | 4.77/4.99 | 5.41/5.42 | 14‡ | *Pnma* | 5.5628(7) | 3.2258(3) | 6.0966(15) | 109.40(3) |

**Table S3** Refinement details of the temperature-dependent laboratory X-ray measurements on CrAs at ambient pressure. Refinements where not all parameters could be refined are marked: * the extinction factor $G_{iso}$ could not be refined; † only one twin component could be refined; ‡ no twin component could be refined.

| $T$ [K] | No. meas. Refl. (obs/all) | $h_{min} \rightarrow h_{max}$ $k_{min} \rightarrow k_{max}$ $l_{min} \rightarrow l_{max}$ | No. sym. ind. Refl. (obs/all) | $R$(int) (obs/all) | $R$ (obs/all) | $wR$ (obs/all) | No. param. | Space group | $a$ [Å] | $b$ [Å] | $c$ [Å] | $V$ [Å³] |
|---|---|---|---|---|---|---|---|---|---|---|---|---|
| 300 | 2183/2509 | −7 → 7<br>−4 → 4<br>−8 → 8 | 169/188 | 6.47/6.51 | 1.95/2.27 | 2.19/2.24 | 14‡ | *Pnma* | 5.6442(10) | 3.4671(6) | 6.2019(10) | 121.37(4) |
| 310 | 2291/2657 | −7 → 7<br>−4 → 4<br>−8 → 8 | 173/192 | 6.05/6.10 | 1.78/2.07 | 1.91/1.95 | 14‡ | *Pnma* | 5.6448(11) | 3.4706(7) | 6.1991(11) | 121.45(4) |
| 320 | 2045/2338 | −7 → 7<br>−4 → 4<br>−8 → 8 | 173/187 | 6.36/6.38 | 2.22/2.42 | 2.46/2.50 | 14‡ | *Pnma* | 5.6475(11) | 3.4714(6) | 6.2010(11) | 121.57(4) |
| 330 | 2248/2563 | −7 → 7<br>−4 → 4<br>−8 → 8 | 173/188 | 6.98/7.00 | 2.26/2.61 | 2.58/2.66 | 14‡ | *Pnma* | 5.6475(11) | 3.4757(6) | 6.2025(11) | 121.75(4) |
| 340 | 2167/2515 | −7 → 7<br>−4 → 4<br>−8 → 8 | 171/190 | 6.08/6.11 | 1.85/2.23 | 2.08/2.17 | 14‡ | *Pnma* | 5.6487(10) | 3.4767(6) | 6.2033(11) | 121.83(4) |
| 350 | 2190/2518 | −7 → 7<br>−4 → 4<br>−8 → 8 | 175/190 | 7.07/7.10 | 2.25/2.49 | 2.42/2.52 | 14‡ | *Pnma* | 5.6501(8) | 3.4794(5) | 6.2054(8) | 121.99(3) |
| 360 | 2126/2491 | −7 → 7<br>−4 → 4<br>−8 → 8 | 171/189 | 6.67/6.70 | 2.15/2.47 | 2.32/2.45 | 14‡ | *Pnma* | 5.6527(9) | 3.4819(5) | 6.2024(9) | 122.07(3) |
| 370 | 2256/2578 | −7 → 7<br>−4 → 4<br>−8 → 8 | 172/189 | 5.92/5.96 | 1.98/2.24 | 2.25/2.33 | 14‡ | *Pnma* | 5.6529(10) | 3.4851(6) | 6.2015(11) | 122.18(4) |
| 380 | 2197/2530 | −7 → 7<br>−4 → 4<br>−8 → 8 | 176/190 | 6.30/6.33 | 2.18/2.41 | 2.38/2.48 | 14‡ | *Pnma* | 5.6543(9) | 3.4860(5) | 6.2046(10) | 122.30(3) |

**Table S3 (cont.)** Refinement details of the temperature-dependent laboratory X-ray measurements on CrAs at ambient pressure. Refinements where not all parameters could be refined are marked: * the extinction factor $G_{iso}$ could not be refined; † only one twin component could be refined; ‡ no twin component could be refined.

| *T* [K] | No. meas. Refl. (obs/all) | $h_{min} \rightarrow h_{max}$ $k_{min} \rightarrow k_{max}$ $l_{min} \rightarrow l_{max}$ | No. sym. ind. Refl. (obs/all) | *R*(int) (obs/all) | *R* (obs/all) | *wR* (obs/all) | No. param. | Space group | *a* [Å] | *b* [Å] | *c* [Å] | *V* [Å³] |
|---|---|---|---|---|---|---|---|---|---|---|---|---|
| 390 | 2348/2663 | −7 → 7<br>−4 → 4<br>−8 → 8 | 178/192 | 5.78/5.82 | 1.94/2.11 | 2.14/2.17 | 14‡ | *Pnma* | 5.6532(9) | 3.4869(5) | 6.2083(9) | 122.38(3) |
| 400 | 2439/2783 | −7 → 7<br>−4 → 4<br>−8 → 8 | 176/191 | 6.56/6.60 | 1.84/2.13 | 1.98/2.11 | 14‡ | *Pnma* | 5.6548(9) | 3.4901(6) | 6.2080(9) | 122.52(3) |
| 290 | 2132/2465 | −7 → 7<br>−4 → 4<br>−8 → 8 | 172/188 | 6.43/6.47 | 2.21/2.49 | 2.35/2.41 | 14‡ | *Pnma* | 5.6434(10) | 3.4630(6) | 6.2027(11) | 121.22(4) |
| 280 | 2320/2656 | −7 → 7<br>−4 → 4<br>−8 → 8 | 172/188 | 6.62/6.66 | 2.24/2.51 | 2.51/2.57 | 14‡ | *Pnma* | 5.6445(9) | 3.4597(6) | 6.1997(10) | 121.07(3) |
| 300 | 2240/2561 | −7 → 7<br>−4 → 4<br>−8 → 8 | 175/189 | 6.50/6.53 | 2.04/2.21 | 2.20/2.23 | 14‡ | *Pnma* | 5.6473(10) | 3.4666(6) | 6.2002(10) | 121.38(4) |
| 310 | 2384/2781 | −7 → 7<br>−4 → 4<br>−8 → 8 | 173/190 | 5.23/5.27 | 1.40/1.92 | 1.58/1.72 | 14‡ | *Pnma* | 5.6477(9) | 3.4693(6) | 6.1995(10) | 121.47(3) |
| 320 | 2382/2782 | −7 → 7<br>−4 → 4<br>−8 → 8 | 174/191 | 5.55/5.59 | 1.81/2.02 | 1.95/1.99 | 14‡ | *Pnma* | 5.6485(9) | 3.4717(6) | 6.1998(10) | 121.58(3) |
| 275 | 2202/2487 | −7 → 7<br>−4 → 4<br>−8 → 8 | 173/186 | 5.70/5.73 | 2.20/2.40 | 2.33/2.40 | 14‡ | *Pnma* | 5.6412(10) | 3.4540(6) | 6.1995(11) | 120.79(4) |

**Table S3 (cont.)** Refinement details of the temperature-dependent laboratory X-ray measurements on CrAs at ambient pressure. Refinements where not all parameters could be refined are marked: * the extinction factor $G_{iso}$ could not be refined; † only one twin component could be refined; ‡ no twin component could be refined.

| $T$ [K] | No. meas. Refl. (obs/all) | $h_{min} \rightarrow h_{max}$ $k_{min} \rightarrow k_{max}$ $l_{min} \rightarrow l_{max}$ | No. sym. ind. Refl. (obs/all) | $R$(int) (obs/all) | $R$ (obs/all) | $wR$ (obs/all) | No. param. | Space group | $a$ [Å] | $b$ [Å] | $c$ [Å] | $V$ [Å³] |
|---|---|---|---|---|---|---|---|---|---|---|---|---|
| 240 | 2566/2961 | −7 → 7 −4 → 4 −8 → 8 | 182/195 | 4.42/4.44 | 1.91/2.25 | 2.46/2.49 | 14‡ | *Pnma* | 5.6172(8) | 3.5776(5) | 6.1449(13) | 123.49(3) |
| 250 | 2599/2964 | −7 → 7 −4 → 4 −8 → 8 | 184/195 | 4.71/4.73 | 2.05/2.17 | 2.89/2.90 | 14‡ | *Pnma* | 5.6183(8) | 3.5757(5) | 6.1488(9) | 123.52(3) |
| 270 | 2582/2919 | −7 → 7 −4 → 4 −8 → 8 | 181/192 | 4.36/4.39 | 1.49/1.74 | 1.76/1.82 | 14‡ | *Pnma* | 5.6445(12) | 3.4579(8) | 6.2078(13) | 121.17(4) |
| 280 | 2517/2927 | −7 → 7 −4 → 4 −8 → 8 | 177/192 | 4.75/4.78 | 1.53/1.74 | 1.78/1.84 | 14‡ | *Pnma* | 5.6450(10) | 3.4590(6) | 6.2001(11) | 121.06(4) |

**Table S4** Refined atom coordinates ($x$, $z$) and isotropic ADP ($U_{iso}$) for Cr and As from the synchrotron X-ray diffraction measurements as function of temperature at ambient pressure.

| | Cr ($x_{Cr}$,¼, $z_{Cr}$) | | As ($x_{As}$,¼, $z_{As}$) | | | |
|---|---|---|---|---|---|---|
| $T$ [K] | $x_{Cr}$ | $z_{Cr}$ | $x_{As}$ | $z_{As}$ | $U_{iso}$[Cr] | $U_{iso}$[As] |
| 275 | 0.00642(3) | 0.20040(3) | 0.20133(20) | 0.57619(18) | 0.00973(3) | 0.00924(20) |
| 270 | 0.00686(4) | 0.20553(4) | 0.20581(3) | 0.58268(3) | 0.00935(3) | 0.00910(3) |
| 260 | 0.00688(4) | 0.20551(4) | 0.20582(3) | 0.58283(3) | 0.00900(3) | 0.00878(3) |
| 250 | 0.00695(3) | 0.20554(3) | 0.20581(3) | 0.58293(19) | 0.00531(3) | 0.00510(20) |
| 240 | 0.00700(3) | 0.20554(3) | 0.20580(18) | 0.58302(16) | 0.00511(3) | 0.00491(17) |
| 220 | 0.00708(3) | 0.20547(3) | 0.20574(17) | 0.58317(15) | 0.00487(20) | 0.00468(16) |
| 200 | 0.00714(3) | 0.20539(3) | 0.20562(18) | 0.58329(16) | 0.00459(3) | 0.00440(17) |
| 185 | 0.00718(3) | 0.20535(3) | 0.20556(16) | 0.58334(15) | 0.00432(18) | 0.00414(15) |
| 170 | 0.00724(3) | 0.20530(3) | 0.20549(15) | 0.58337(14) | 0.00409(18) | 0.00390(15) |
| 155 | 0.00728(3) | 0.20522(3) | 0.20540(16) | 0.58340(15) | 0.00399(19) | 0.00380(16) |
| 140 | 0.00732(3) | 0.20514(3) | 0.20532(18) | 0.58342(17) | 0.00386(3) | 0.00366(17) |
| 125 | 0.00736(3) | 0.20508(3) | 0.20526(17) | 0.58343(15) | 0.00364(19) | 0.00342(16) |
| 110 | 0.00739(3) | 0.20504(3) | 0.20518(16) | 0.58347(15) | 0.00349(18) | 0.00328(15) |
| 95 | 0.00740(20) | 0.20493(20) | 0.20510(15) | 0.58348(14) | 0.00329(17) | 0.00307(14) |
| 80 | 0.00745(3) | 0.20491(3) | 0.20499(20) | 0.58347(19) | 0.00322(3) | 0.00300(20) |
| 65 | 0.00751(18) | 0.20480(17) | 0.20492(13) | 0.58348(11) | 0.00284(15) | 0.00259(12) |
| 50 | 0.00752(18) | 0.20474(17) | 0.20486(13) | 0.58348(12) | 0.00271(15) | 0.00244(13) |
| 35 | 0.00753(5) | 0.20473(5) | 0.20486(4) | 0.58347(3) | 0.00324(4) | 0.00298(3) |

**Table S5** Refined atom coordinates (*x*, *z*) and isotropic ADP ($U_{iso}$) for Cr and As from the synchrotron X-ray diffraction measurements as function of pressure at room temperature.

| | Cr ($x_{Cr}$,¼, $z_{Cr}$) | | As ($x_{As}$,¼, $z_{As}$) | | | |
|---|---|---|---|---|---|---|
| *p* [GPa] | $x_{Cr}$ | $z_{Cr}$ | $x_{As}$ | $z_{As}$ | $U_{iso}$[Cr] | $U_{iso}$[As] |
| 1.03 | 0.00629(17) | 0.19832(18) | 0.19901(11) | 0.57407(11) | 0.0163(5) | 0.0152(3) |
| 2.53 | 0.00676(19) | 0.19605(19) | 0.19680(13) | 0.57242(12) | 0.0158(5) | 0.0154(3) |
| 3.13 | 0.0067(3) | 0.1956(3) | 0.19610(15) | 0.57182(13) | 0.0156(4) | 0.0155(3) |
| 4.45 | 0.0070(7) | 0.1944(3) | 0.1948(4) | 0.57133(17) | 0.0170(9) | 0.0158(6) |
| 6.05 | 0.0084(8) | 0.1936(4) | 0.1941(5) | 0.57039(19) | 0.016(11) | 0.0157(8) |
| 7.32 | 0.00742(18) | 0.1930(3) | 0.19293(12) | 0.56996(14) | 0.0137(4) | 0.0132(3) |
| 8.09 | 0.0078(3) | 0.1926(3) | 0.19252(14) | 0.56959(15) | 0.0156(4) | 0.0156(3) |
| 9.46 | 0.0075(3) | 0.1920(3) | 0.19160(13) | 0.56928(16) | 0.0135(5) | 0.0134(4) |

**Table S6** Refined atom coordinates ($x$, $z$) and isotropic ADP ($U_{iso}$) for Cr and As from the laboratory X-ray diffraction measurements as function of temperature at ambient pressure.

| | Cr ($x_{Cr}$,¼, $z_{Cr}$) | | As ($x_{As}$,¼, $z_{As}$) | | | |
|---|---|---|---|---|---|---|
| $T$ [K] | $x_{Cr}$ | $z_{Cr}$ | $x_{As}$ | $z_{As}$ | $U_{iso}$[Cr] | $U_{iso}$[As] |
| 300 | 0.00592(13) | 0.20325(12) | 0.20404(9) | 0.57776(7) | 0.0049(3) | 0.0044(2) |
| 310 | 0.00602(13) | 0.20307(12) | 0.20383(10) | 0.57772(7) | 0.0048(3) | 0.0043(2) |
| 320 | 0.00596(16) | 0.20303(15) | 0.20366(11) | 0.57768(8) | 0.0052(3) | 0.0047(3) |
| 330 | 0.00603(15) | 0.20290(14) | 0.20369(11) | 0.57764(8) | 0.0057(2) | 0.0053(3) |
| 340 | 0.00625(16) | 0.20264(15) | 0.20357(12) | 0.57751(9) | 0.0056(3) | 0.0051(2) |
| 350 | 0.00613(16) | 0.20258(15) | 0.20346(12) | 0.57743(9) | 0.0056(3) | 0.0051(3) |
| 360 | 0.00610(14) | 0.20226(13) | 0.20328(11) | 0.57737(8) | 0.0059(3) | 0.0055(3) |
| 370 | 0.00620(17) | 0.20222(15) | 0.20311(12) | 0.57718(9) | 0.0062(3) | 0.0058(3) |
| 380 | 0.00611(16) | 0.20208(15) | 0.20305(12) | 0.57712(9) | 0.0064(3) | 0.0059(3) |
| 390 | 0.00616(13) | 0.20149(11) | 0.20243(9) | 0.57703(7) | 0.0066(3) | 0.0062(2) |
| 400 | 0.00627(15) | 0.20152(13) | 0.20264(11) | 0.57684(8) | 0.0067(3) | 0.0062(2) |
| 290 | 0.00626(16) | 0.20128(14) | 0.20241(11) | 0.57674(9) | 0.0046(3) | 0.0044(3) |
| 280 | 0.00625(16) | 0.20116(15) | 0.20218(12) | 0.57651(9) | 0.0049(3) | 0.0044(3) |
| 300 | 0.00630(14) | 0.20152(13) | 0.20236(10) | 0.57690(8) | 0.0046(3) | 0.0042(2) |
| 310 | 0.00622(11) | 0.20150(10) | 0.20245(8) | 0.57697(6) | 0.0046(2) | 0.0042(2) |
| 320 | 0.00627(13) | 0.20175(12) | 0.20274(9) | 0.57717(7) | 0.0048(3) | 0.0045(2) |
| 275 | 0.00622(15) | 0.20110(14) | 0.20182(11) | 0.57640(8) | 0.0040(3) | 0.0038(3) |
| 240 | 0.00686(15) | 0.20602(14) | 0.20618(10) | 0.58287(8) | 0.0046(3) | 0.0040(3) |
| 250 | 0.00690(16) | 0.20624(16) | 0.20636(11) | 0.58284(9) | 0.0048(3) | 0.0044(3) |
| 270 | 0.00652(11) | 0.20057(10) | 0.20156(7) | 0.57631(6) | 0.0050(2) | 0.0045(2) |
| 280 | 0.00625(12) | 0.20081(11) | 0.20183(8) | 0.57650(6) | 0.0049(3) | 0.0045(2) |

**Table S7** Key to the denotation of the interatomic distances in CrAs. Symmetry code: (I) *x*, *y*, *z*; (II) *x*+½, −*y*+½, −*z*+½; (III) −*x*+½, −*y*, *z*+½; (IV) −*x*, *y*+½, −*z*; (V) −*x*, −*y*, −*z*; (VI) *x*+½, *y*, −*z*+½; (VII) *x*, −*y*+½, *z*; (VIII) −*x*+½, *y*+½, *z*+½. In the MnP-type structures with both Cr and As occupying Wyckoff 4*c* positions at (*x*, ¼, *z*), the sites (I)/(VII), (II)/(VI), (III)/(VIII) and (IV)/(V) are pairwise degenerated. Symmetrically equivalent position outside of the unit cell are marked by the according translation vector, e.g. “I (0,1,0)” for the position *x*, *y*+1, *z*. The color markings correspond to the ones shown in Figure 7.

| | Cr–Cr | | | Cr–As | | | |
|---|---|---|---|---|---|---|---|
| Distance shorthand | ■ Cr$^{I}$–Cr$^{I}$ | ■ Cr$^{I}$–Cr$^{II}$ | ■ Cr$^{I}$–Cr$^{IV}$ | ■ Cr$^{I}$–As$^{I}$ | ■ Cr$^{I}$–As$^{II}$ | ■ Cr$^{I}$–As$^{III}$ | ■ Cr$^{I}$–As$^{IV}$ |
| Distances from Cr$^{I}$ | Cr$^{I}$–Cr$^{I\ (0,1,0)}$<br>Cr$^{I}$–Cr$^{I\ (0,-1,0)}$ | Cr$^{I}$–Cr$^{II}$<br>Cr$^{I}$–Cr$^{II\ (0,-1,0)}$ | Cr$^{I}$–Cr$^{IV\ (0,0,-1)}$<br>Cr$^{I}$–Cr$^{IV\ (0,-1,-1)}$ | Cr$^{I}$–As$^{I}$ | Cr$^{I}$–As$^{II\ (-1,0,0)}$ | Cr$^{I}$–As$^{III\ (0,0,-1)}$<br>Cr$^{I}$–As$^{III\ (0,1,-1)}$ | Cr$^{I}$–As$^{IV\ (0,0,1)}$<br>Cr$^{I}$–As$^{IV\ (0,1,1)}$ |
| Equivalent distances | Cr$^{II}$–Cr$^{II\ (0,1,0)}$<br>Cr$^{II}$–Cr$^{II\ (0,-1,0)}$<br>Cr$^{III}$–Cr$^{III\ (0,1,0)}$<br>Cr$^{III}$–Cr$^{III\ (0,-1,0)}$<br>Cr$^{IV}$–Cr$^{IV\ (0,1,0)}$<br>Cr$^{IV}$–Cr$^{IV\ (0,-1,0)}$ | Cr$^{III}$–Cr$^{IV}$<br>Cr$^{III}$–Cr$^{IV\ (0,-1,0)}$ | Cr$^{II}$–Cr$^{III}$<br>Cr$^{II}$–Cr$^{III\ (0,-1,0)}$ | Cr$^{II}$–As$^{II}$<br>Cr$^{III}$–As$^{III}$<br>Cr$^{IV}$–As$^{IV}$ | Cr$^{II}$–As$^{I}$<br>Cr$^{III}$–As$^{IV\ (1,0,1)}$<br>Cr$^{IV}$–As$^{III\ (0,0,-1)}$ | Cr$^{II}$–As$^{IV\ (1,0,1)}$<br>Cr$^{II}$–As$^{IV\ (1,1,1)}$<br>Cr$^{III}$–As$^{I}$<br>Cr$^{III}$–As$^{I\ (0,1,0)}$<br>Cr$^{IV}$–As$^{II\ (-1,0,0)}$<br>Cr$^{IV}$–As$^{II\ (-1,1,0)}$ | Cr$^{II}$–As$^{III\ (0,0,-1)}$<br>Cr$^{II}$–As$^{III\ (0,1,-1)}$<br>Cr$^{III}$–As$^{II\ (0,0,1)}$<br>Cr$^{III}$–As$^{II\ (0,-1,1)}$<br>Cr$^{IV}$–As$^{I\ (0,0,-1)}$<br>Cr$^{IV}$–As$^{I\ (0,-1,-1)}$ |

**Table S8** Interatomic Cr–Cr and Cr–As distances in CrAs calculated from the synchrotron X-ray diffraction measurements as function of temperature at ambient pressure. The denotation of the distances follows **Table S7**.

| | Cr–Cr | | | Cr–As | | | |
|---|---|---|---|---|---|---|---|
| *T* [K] | ■ $Cr^{I}$–$Cr^{I}$ [Å] | ■ $Cr^{I}$–$Cr^{II}$ [Å] | ■ $Cr^{I}$–$Cr^{IV}$ [Å] | ■ $Cr^{I}$–$As^{I}$ [Å] | ■ $Cr^{I}$–$As^{II}$ [Å] | ■ $Cr^{I}$–$As^{III}$ [Å] | ■ $Cr^{I}$–$As^{IV}$ [Å] |
| 275 | 3.4520(2) | 2.8878(2) | 3.0271(2) | 2.5772(2) | 2.4303(2) | 2.50846(16) | 2.50465(16) |
| 270 | 3.5731(2) | 2.8630(3) | 3.0968(3) | 2.5752(3) | 2.4506(3) | 2.52406(19) | 2.5135(2) |
| 260 | 3.5753(2) | 2.8622(3) | 3.0967(3) | 2.5754(3) | 2.4506(3) | 2.52406(18) | 2.51359(19) |
| 250 | 3.5772(2) | 2.8614(2) | 3.0967(2) | 2.5747(2) | 2.4507(2) | 2.52398(16) | 2.51357(16) |
| 240 | 3.5784(2) | 2.8607(2) | 3.0967(2) | 2.57448(19) | 2.45072(19) | 2.52380(14) | 2.51357(15) |
| 220 | 3.5805(2) | 2.8593(2) | 3.09564(19) | 2.57413(18) | 2.45039(18) | 2.52342(14) | 2.51349(14) |
| 200 | 3.5815(2) | 2.8583(2) | 3.0946(2) | 2.57410(19) | 2.45042(19) | 2.52313(14) | 2.51315(15) |
| 185 | 3.5826(4) | 2.8578(3) | 3.0937(2) | 2.5734(2) | 2.4501(2) | 2.52315(19) | 2.51306(19) |
| 170 | 3.5831(2) | 2.8572(3) | 3.0929(2) | 2.5729(2) | 2.44994(19) | 2.52288(15) | 2.51296(14) |
| 155 | 3.5835(2) | 2.8564(3) | 3.0917(2) | 2.5725(2) | 2.4495(2) | 2.52257(15) | 2.51282(14) |
| 140 | 3.5839(2) | 2.8555(3) | 3.0907(2) | 2.5722(2) | 2.4490(2) | 2.52227(16) | 2.51269(16) |
| 125 | 3.5837(2) | 2.8554(3) | 3.0899(2) | 2.5721(2) | 2.4490(2) | 2.52206(15) | 2.51259(15) |
| 110 | 3.5840(2) | 2.8552(3) | 3.0891(2) | 2.5718(2) | 2.4490(2) | 2.52206(15) | 2.51235(14) |
| 95 | 3.5842(2) | 2.8550(3) | 3.0878(2) | 2.5719(2) | 2.44851(19) | 2.52192(14) | 2.51236(14) |
| 80 | 3.5838(2) | 2.8544(3) | 3.0871(2) | 2.5710(2) | 2.4485(2) | 2.52173(17) | 2.51187(17) |
| 65 | 3.5843(2) | 2.85401(17) | 3.08560(19) | 2.57077(19) | 2.44795(16) | 2.52139(12) | 2.51208(12) |
| 50 | 3.5844(2) | 2.85383(17) | 3.08491(19) | 2.57059(19) | 2.44775(16) | 2.52137(12) | 2.51204(12) |
| 35 | 3.5844(2) | 2.8530(4) | 3.0854(3) | 2.5711(3) | 2.4477(3) | 2.5211(2) | 2.5122(2) |

**Table S9** Interatomic Cr–Cr and Cr–As distances in CrAs calculated from the synchrotron X-ray diffraction measurements as function of pressure at room temperature. The denotation of the distances follows Table S7.

| | Cr–Cr | | | Cr–As | | | |
|---|---|---|---|---|---|---|---|
| *p* [GPa] | ■ $Cr^{I}$–$Cr^{I}$ [Å] | ■ $Cr^{I}$–$Cr^{II}$ [Å] | ■ $Cr^{I}$–$Cr^{IV}$ [Å] | ■ $Cr^{I}$–$As^{I}$ [Å] | ■ $Cr^{I}$–$As^{II}$ [Å] | ■ $Cr^{I}$–$As^{III}$ [Å] | ■ $Cr^{I}$–$As^{IV}$ [Å] |
| 1.03 | 3.390(2) | 2.8854(17) | 2.9846(15) | 2.5666(14) | 2.4153(13) | 2.4930(12) | 2.4887(12) |
| 2.53 | 3.342(2) | 2.8854(18) | 2.9431(16) | 2.5577(15) | 2.4037(14) | 2.4790(13) | 2.4785(13) |
| 3.13 | 3.326(2) | 2.884(3) | 2.929(2) | 2.550(2) | 2.3981(19) | 2.4750(16) | 2.4727(15) |
| 4.45 | 3.3003(16) | 2.879(7) | 2.906(2) | 2.544(3) | 2.391(4) | 2.465(4) | 2.464(3) |
| 6.05 | 3.2734(14) | 2.875(7) | 2.884(2) | 2.531(3) | 2.386(4) | 2.451(4) | 2.459(3) |
| 7.32 | 3.2539(12) | 2.874(2) | 2.868(4) | 2.528(4) | 2.380(2) | 2.4506(13) | 2.4493(18) |
| 8.09 | 3.2432(10) | 2.873(2) | 2.860(2) | 2.524(2) | 2.3779(18) | 2.4463(14) | 2.4470(15) |
| 9.46 | 3.2258(6) | 2.8698(18) | 2.845(2) | 2.518(2) | 2.3721(17) | 2.4418(12) | 2.4385(13) |

**Table S10** Interatomic Cr–Cr and Cr–As distances in CrAs calculated from the laboratory X-ray diffraction measurements as function of temperature at ambient pressure. The denotation of the distances follows Table S7.

| | Cr–Cr | | | Cr–As | | | |
|---|---|---|---|---|---|---|---|
| *T* [K] | ■ $Cr^{I}$–$Cr^{I}$ [Å] | ■ $Cr^{I}$–$Cr^{II}$ [Å] | ■ $Cr^{I}$–$Cr^{IV}$ [Å] | ■ $Cr^{I}$–$As^{I}$ [Å] | ■ $Cr^{I}$–$As^{II}$ [Å] | ■ $Cr^{I}$–$As^{III}$ [Å] | ■ $Cr^{I}$–$As^{IV}$ [Å] |
| 300 | 3.4671(12) | 2.8854(15) | 3.0428(12) | 2.5781(11) | 2.4326(11) | 2.5105(9) | 2.5070(9) |
| 310 | 3.4706(14) | 2.8858(15) | 3.0424(11) | 2.5782(11) | 2.4332(10) | 2.5125(9) | 2.5066(8) |
| 320 | 3.4714(12) | 2.8856(16) | 3.0493(13) | 2.5779(13) | 2.4345(12) | 2.5122(10) | 2.5065(9) |
| 330 | 3.4757(12) | 2.8853(17) | 3.0525(13) | 2.5779(13) | 2.4358(13) | 2.5133(10) | 2.5079(10) |
| 340 | 3.4767(12) | 2.8858(15) | 3.0535(12) | 2.5797(12) | 2.4362(11) | 2.5134(9) | 2.5079(9) |
| 350 | 3.4794(10) | 2.8857(15) | 3.0582(12) | 2.5795(12) | 2.4379(12) | 2.5144(9) | 2.5085(9) |
| 360 | 3.4819(10) | 2.8868(15) | 3.0586(13) | 2.5788(12) | 2.4386(12) | 2.5147(9) | 2.5094(9) |
| 370 | 3.4851(12) | 2.8862(15) | 3.0618(13) | 2.5786(12) | 2.4388(12) | 2.5164(9) | 2.5088(9) |
| 380 | 3.4860(10) | 2.8866(15) | 3.0643(12) | 2.5796(12) | 2.4399(12) | 2.5173(10) | 2.5091(9) |
| 390 | 3.4869(10) | 2.8860(14) | 3.0663(11) | 2.5808(11) | 2.4408(10) | 2.5171(8) | 2.5098(8) |
| 400 | 3.4901(12) | 2.8864(13) | 3.0689(11) | 2.5808(12) | 2.4408(11) | 2.5184(9) | 2.5106(9) |
| 290 | 3.4630(12) | 2.8857(16) | 3.0394(13) | 2.5786(13) | 2.4319(12) | 2.5095(10) | 2.5061(9) |
| 280 | 3.4597(12) | 2.8865(15) | 3.0363(13) | 2.5765(12) | 2.4309(12) | 2.5096(12) | 2.5053(9) |
| 300 | 3.4666(12) | 2.8869(15) | 3.0420(12) | 2.5774(11) | 2.4344(11) | 2.5116(11) | 2.5061(9) |
| 310 | 3.4693(12) | 2.8872(12) | 3.0424(10) | 2.5781(10) | 2.4339(9) | 2.5124(09) | 2.5068(8) |
| 320 | 3.4717(12) | 2.8869(13) | 3.0457(11) | 2.5786(11) | 2.4351(10) | 2.5122(10) | 2.5071(8) |
| 275 | 3.4540(12) | 2.8850(15) | 3.0339(12) | 2.5751(12) | 2.4307(11) | 2.5086(9) | 2.5025(9) |
| 240 | 3.5776(10) | 2.8601(14) | 3.1010(13) | 2.5722(13) | 2.4503(12) | 2.5240(8) | 2.5129(8) |
| 250 | 3.5757(10) | 2.8602(15) | 3.1041(13) | 2.5725(13) | 2.4516(12) | 2.5234(9) | 2.5128(9) |
| 270 | 3.4579(16) | 2.8882(14) | 3.0324(12) | 2.5793(11) | 2.4325(10) | 2.5098(9) | 2.5074(8) |
| 280 | 3.4590(12) | 2.8877(13) | 3.0326(11) | 2.5777(11) | 2.4309(10) | 2.5101(8) | 2.5054(7) |

### Appendix A. Calculation of distance distortion and angular distortion

Within this work and as part of the overall study of the CrAs structure, the structural distortion of two coordination polyhedra (the [$CrAs_6$] octrahedron and the [$AsCr_6$] trigonal prism) was quantified to measure the effect of temperature and pressure. While a number of different distortion parameters exists for the octahedron – and, in general, for the "ideal" polyhedra – [Baur, 1974; Robinson, Gibbs and Ribbe, 1970; Brown & Shannon, 1973; Tarakina *et al*., 2003; Wang & Liebau, 2007; Guionneau *et al*., 2002], those are not easily suitable to be applied to the non-"ideal" trigonal prism. In order to keep the calculated distortion for the octahedron and the trigonal prism analogous, we use distortion parameters that can be seen as generalized adaptions of previously reported distortion parameters:

The distance distortion $\delta_D$ is defined similar to the one introduced by Baur [1974]:

$$\delta_{\mathrm{D}} = \frac{1}{n} \cdot \sqrt{\sum_{i=1}^{n} \left( \frac{d_i - \bar{d}}{\bar{d}} \right)^2}$$

with the different intrapolyhedral center-vortex distances $d$, the average distance $\bar{d}$ and the number of distances $n$.

The angular distortion $\delta_A$ is similarly based on the angular distortion introduced by Baur [1974], taking into account a basic modification made by Zheng *et al*. [2019] to account for the occurrence of essentially different values in the structure:

$$\delta_{\mathrm{A}} = \frac{1}{m} \cdot \sqrt{\sum_{i=1}^{p} \sum_{j=1}^{q_i} \left( \frac{\varphi_{i,j} - \bar{\varphi}_i}{\bar{\varphi}_i} \right)^2}$$

The total number $m$ of intrapolyhedral vortex–center–vortex angles $\varphi$ is divided into $p$ groups of angles that are degenerated in the considered reference structure, each group (index $i$) comprising $q_i$ angles with average $\bar{\varphi}_i$. The details for the two considered coordination polyhedra are shown in **Tables S11** and **S12**.

**Table S11** Details for the calculation of the angular distortion $\delta_A$ of the distorted $[CrAs_6]$ octahedron.

| $[CrAs_6]$ | Ideal Structure (Octahedron) | | Real Structure |
|---|---|---|---|
| | 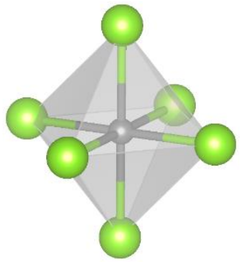 | | 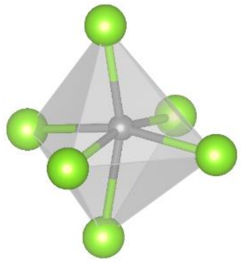 |
| **Angle Group *i*** | ***i* = 1** | ***i* = 2** | |
| **Visualization** | 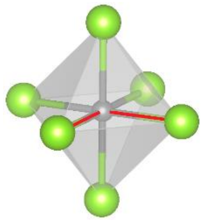 | 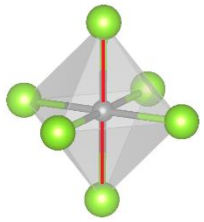 | |
| **Degeneracy $q_i$** | $q_1 = 12$ | $q_2 = 3$ | |

**Table S12** Details for the calculation of the angular distortion $\delta_A$ of the distorted $[AsCr_6]$ trigonal prism.

| $[AsCr_6]$ | Ideal Structure (Trigonal Prism) | | Real Structure |
|---|---|---|---|
| | 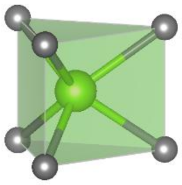 | | 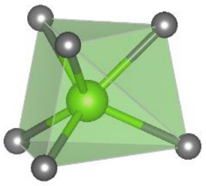 |
| **Angle Group *i*** | ***i* = 1** | ***i* = 2** | ***i* = 3** |
| **Visualization** | 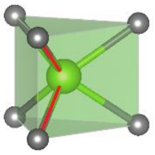 | 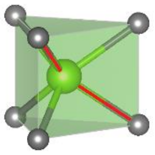 | 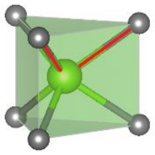 |
| **Degeneracy $q_i$** | $q_1 = 3$ | $q_2 = 6$ | $q_3 = 6$ |

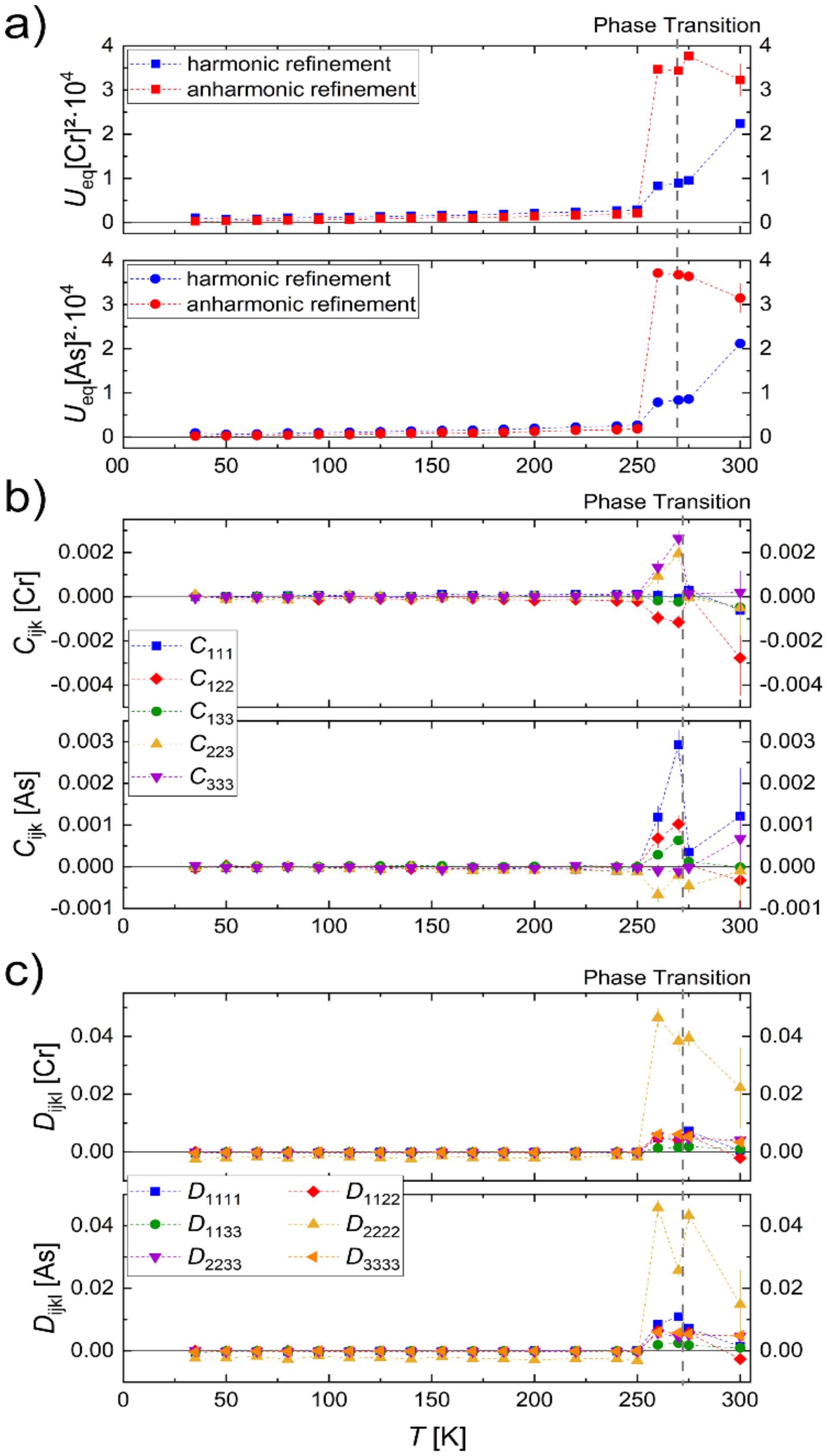


**Figure S1** The harmonic ADP $U_{eq}^2$ (a) and the anharmonic ADP of third order $C_{ijk}$ (b, c) and of fourth order $D_{ijkl}$ (d, e) for the synchrotron X-ray measurements. Only the significant parameters are shown as function of temperature at ambient pressure. The dash lines are guides for the eye.

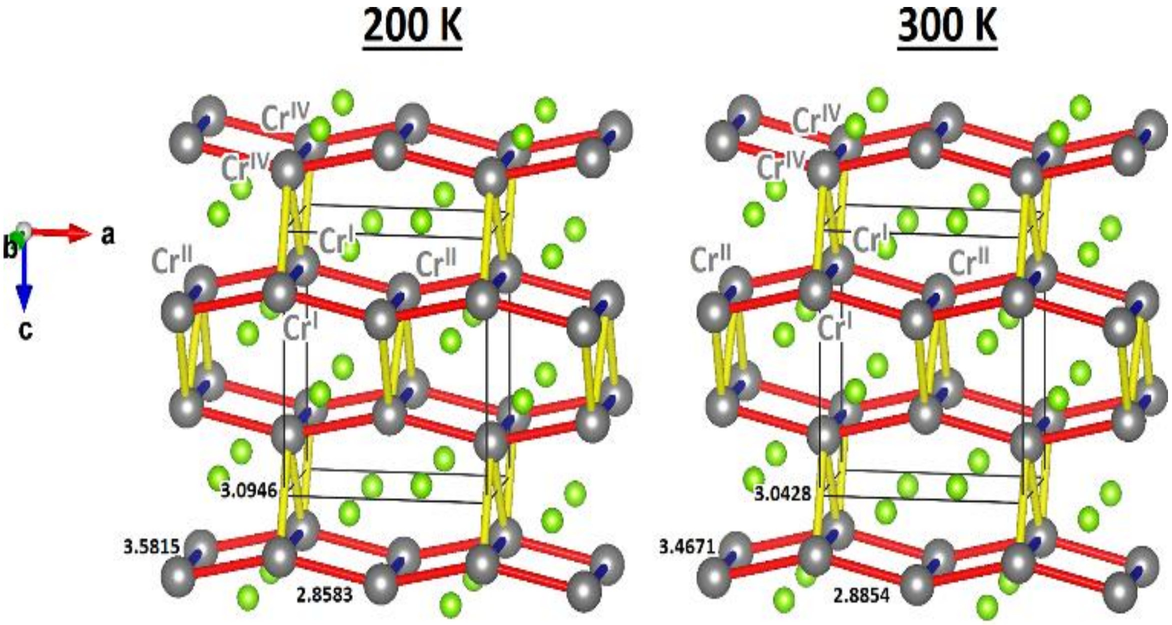


**Figure S2** Overview of the Cr–Cr distances in the crystal structure of CrAs below the phase transition (200 K, *left*) and above the phase transition (300 K, *right*). The Cr and As atoms are shown in dark gray and green, respectively. The distance values are given in [Å].

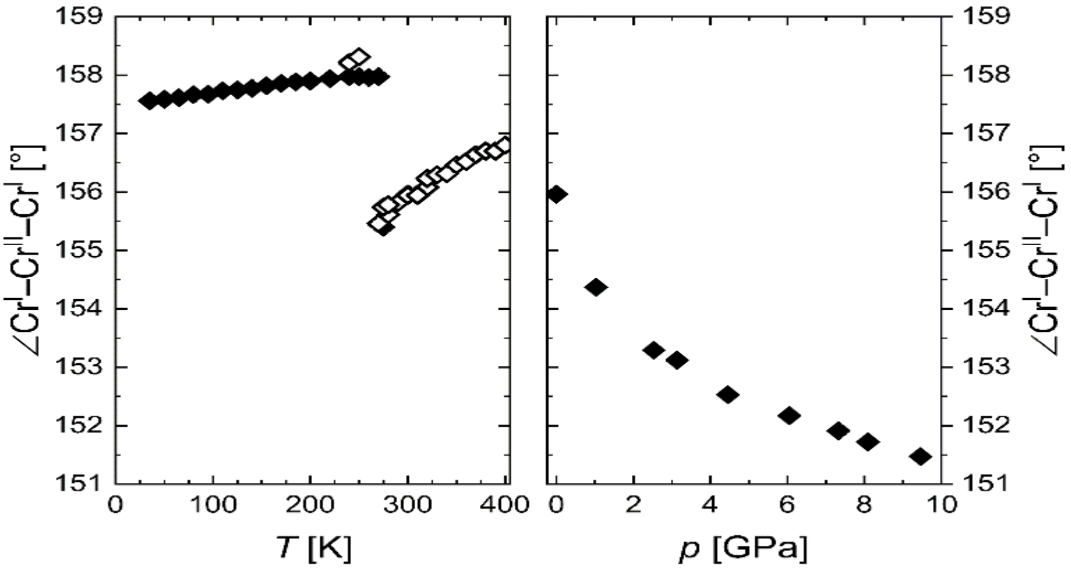


**Figure S3** The angle $\angle Cr^{I}–C^{II}–Cr^{I}$ as a function of temperature (*left*) and pressure (*right*).

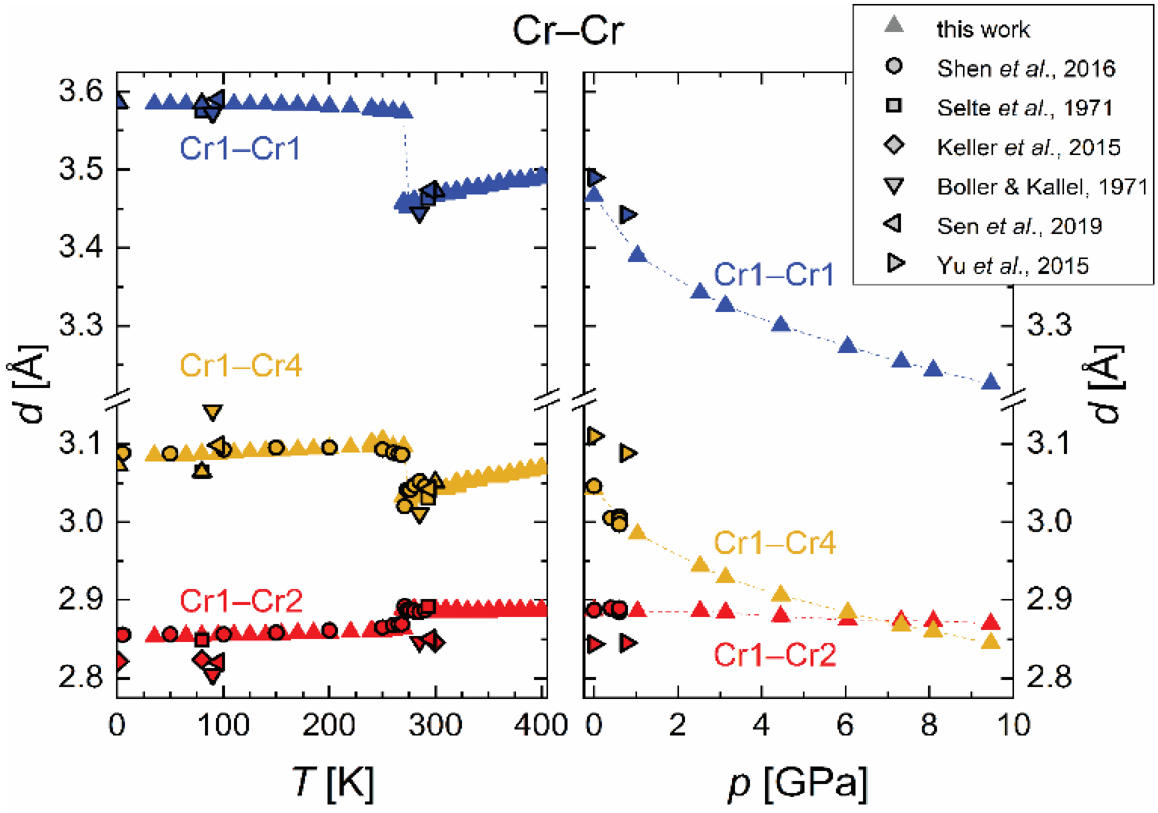


**Figure S4** Comparison of the measured Cr–Cr distances as function of temperature (*left*) and pressure (*right*) for CrAs within this work and reported in the literature. The dash lines are guides for the eye.
